# XI-006 induces potent p53-independent apoptosis in Ewing sarcoma

**DOI:** 10.1038/srep11465

**Published:** 2015-06-22

**Authors:** Kathleen I. Pishas, Alaknanda Adwal, Susan J. Neuhaus, Mark T. Clayer, Gelareh Farshid, Alexander H. Staudacher, David F. Callen

**Affiliations:** 1 Sarcoma Research Group, Discipline of Medicine, University of Adelaide, Adelaide, Australia; 2 Cancer Therapeutics Laboratory, Discipline of Medicine, University of Adelaide, Adelaide, Australia; 3 Department of Surgery, Royal Adelaide Hospital and University of Adelaide, Adelaide, Australia; 4 Department of Orthopaedics and Trauma, Royal Adelaide Hospital, Adelaide, Australia; 5 Division of Tissue Pathology, SA Pathology, Adelaide, Australia; 6 Translational Oncology Laboratory, Centre for Cancer Biology, SA Pathology, Adelaide, Australia; 7 School of Medicine, University of Adelaide, Adelaide, Australia

## Abstract

There is an imperious need for the development of novel therapeutics for the treatment of Ewing sarcoma, the second most prevalent solid bone tumour observed in children and young adolescents. Recently, a 4-nitrobenzofuroxan derivative, XI-006 (NSC207895) was shown to diminish MDM4 promoter activity in breast cancer cell lines. As amplification of MDM4 is frequently observed in sarcomas, this study examined the therapeutic potential of XI-006 for the treatment of Ewing and osteosarcoma. XI-006 treatment of Ewing and osteosarcoma cell lines (n = 11) resulted in rapid and potent apoptosis at low micro-molar concentrations specifically in Ewing sarcoma cell lines (48 hr IC50 0.099–1.61 μM). Unexpectedly, apoptotic response was not dependent on *MDM4* mRNA/protein levels or *TP53* status. Alkaline/neutral comet and γH2AX immunofluorescence assays revealed that the cytotoxic effects of XI-006 could not be attributed to the induction of DNA damage. RNA expression analysis revealed that the mechanism of action of XI-006 could be accredited to the inhibition of cell division and cycle regulators such as *KIF20A* and *GPSM2*. Finally, potent synergy between XI-006 and olaparib (PARP inhibitor) were observed due to the down-regulation of *Mre11*. Our findings suggest that XI-006 represents a novel therapeutic intervention for the treatment of Ewing sarcoma.

Sarcomas are a group of rare malignancies that affect approximately 200,000 individuals worldwide each year^1^. Exemplifying the heterogeneous nature of this malignancy type, approximately 50 distinct histological subtypes of sarcoma have been identified to date, ranging from indolent to highly invasive and metastatic^2^. The introduction of cytotoxic chemotherapeutic agents such as doxorubicin in the 1960’s for chemo-sensitive subtypes was a paradigm shift in oncology practice, however current multi-agent chemotherapeutic regimens are associated with significant cumulative and late toxicities. With the exception of gastrointestinal stromal tumours (GIST), limited progress in the management of sarcomas has been achieved over the past few decades years. For this reason, the advent of novel and targeted therapeutics with favourable efficacy and toxicity profiles are eagerly awaited, especially for those 20–40% of patients with non-responding, unresectable or metastatic disease.

Cancer is a multifaceted process that can arise due to the activation of proto-oncogenes and/or inactivation of tumour suppressor genes. The development of novel anti-cancer agents, specifically those focused on targeting oncogene addiction has undergone a dramatic renaissance over the past decade. One particular oncogene which has gained significant interest is *MDM4* (*Mouse Double Minute 4*), a structural homologue of *MDM2*, thought to promote tumourigenesis via its ability to inhibit the tumour suppressor function of *TP53*^3^. In keeping with this hypothesis, amplification and/or overexpression of *MDM4* has been documented across a wide spectrum of tumours including cutaneous melanoma (68.5%)^4^, retinoblastoma (65%)^5^, head and neck squamous carcinoma (50%)^6^ , breast (19%)^3^ and sarcoma (17%)^7^,^8^. In particular, MDM4 copy number gain was documented in 54% of conventional, intramedullary, high-grade osteosarcomas and 33% of parosteal osteosarcomas^9^. Furthermore, amplification of MDM4 defined as >3 fold was shown to be a distinctive attribute of Ewing, synovial and osteosarcomas, with amplification observed in 50%, 44% and 35% of tumour samples respectively^8^. Prevailing evidence suggests that MDM4 primarily represses the transcriptional activity of p53 by binding its trans-activation domain. However, although displaying no intrinsic E3 ubiquitin ligase activity, MDM4 can also regulate p53 stability by promoting MDM2-mediated degradation^10^,^11^.

Owing to the prevalence of MDM4 genomic amplification/mRNA overexpression in human cancers, several strategies aimed at inhibiting the oncogenic activity of MDM4 have been explored. Although a selective MDM4 small-molecule inhibitor does not currently exist, the first reported p53-MDM4 antagonist, SJ-172550, did exhibit cytotoxicity in retinoblastoma cells^12^. However, the thiol reactivity of SJ-172550 precludes its chemical scaffold from further development^13^. Recently, a peptide antagonist of the p53-MDM4 interaction, designated SAH-p53-8 has been developed. This stapled peptide possesses substantially improved pharmacokinetic profiles compared to non-stapled peptide counterparts, and has nano-molar binding affinity to the N-terminal p53-binding pocket of both MDM2 and MDM4^14^. However, the bioavailability of stapled peptides and their potential as therapeutic agents has been questioned. Small molecules are considered more desirable for cancer therapy as their cellular uptake is dependent on passive diffusion, whereas stapled peptides such as SAH-p53-8 require pinocytosis, which is less effective^15^. Indeed, this is highlighted by the fact that high concentrations of SAH-p53-8 (15–30 μM) were required to induce significant cytotoxicity in melanoma cells *in vitro*, uptake was attenuated in the presence of serum, and complete regression of xenograft tumours was not achieved^4^,^16^. Given that aberrant transcription of MDM4 can be attributed to its overexpression in cancer^17^, Wang and colleagues employed a high-throughput drug screening strategy to identify small molecules that could mitigate MDM4 promoter activity. A 4-nitrobenzofuroxan derivative, designated XI-006 (NSC207895) was identified and was shown to repress MDM4 promoter activity resulting in decreased *MDM4* mRNA and protein expression and cell viability in MDM4 amplified breast cancer cell lines^18^.

To our knowledge, no studies have hitherto directly addressed whether repression of MDM4 activity can represent a novel therapeutic strategy for the treatment of sarcomas. In particular, as MDM4 amplification is a characteristic of both Ewing and osteosarcoma, this study has examined the biological effects of XI-006 both as a single agent and in combination with standard chemotherapeutic agents and olaparib (PARP inhibitor) in a comprehensive panel of Ewing and osteosarcoma cell lines *in vitro*. Specifically, treatment of Ewing sarcoma cell lines resulted in potent apoptosis that was remarkably not dependent on *MDM4* mRNA or protein levels or *TP53* status.

## Results

### MDM4 protein is overexpressed in sarcomas

**Figure 1:**
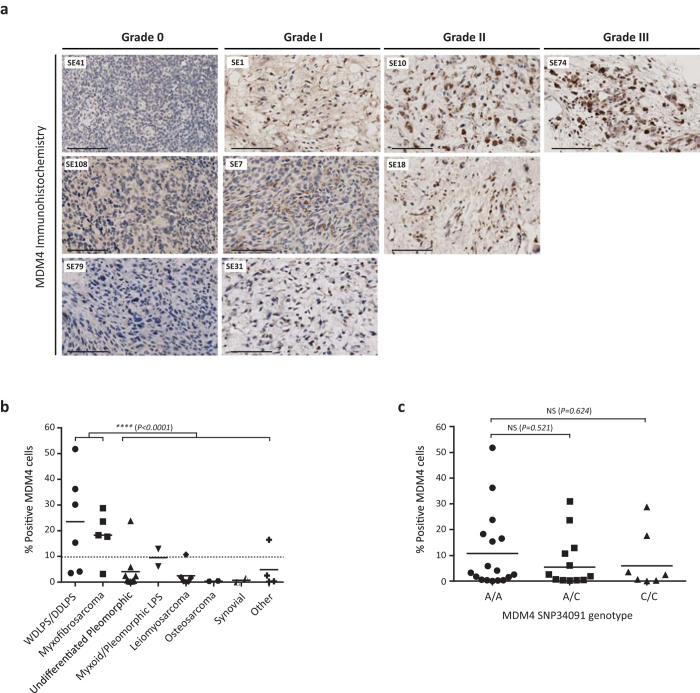
MDM4 protein is overexpressed in sarcomas a) Representative images of sarcoma MDM4 immunohistochemical staining. MDM4 grading determined from the average number of MDM4 positive cells from four fields of view, Grade 0 (<10% positive cells), Grade I (11–25% positive cells), Grade II (26–50% positive cells), Grade III (>51% positive cells) Scale bar=100uM. (b) Correlation between sarcoma pathology and percentage positive MDM4 cells determined from immunohistochemical analysis. Asterisk denotes statistical significance in MDM4 expression (****P < 0.0001). (c) Lack of correlation between MDM4 SNP34091 genotype (AA, AC, CC) and percentage positive MDM4 cells determined from immunohistochemical analysis.

The majority of studies that have evaluated sarcoma MDM4 expression levels have done so through quantification of mRNA. As *MDM4* mRNA expression was recently shown not to correlate with protein expression in freshly isolated human melanomas^4^, these previous studies may have grossly underestimated the frequency of MDM4 protein expression in sarcomas. Indeed, *MDM4* mRNA overexpression was not observed in our previous cohort of 24 sarcoma tissues^19^. As such, MDM4 protein expression in a cohort of 36 sarcoma samples of varying histopathology was determined through immunohistochemical analysis (IHC). Although MDM4 expression was very low to undetectable (<10% MDM4 positive cells) in 24/36 (66.7%) of tumour samples, strong positive staining was observed in 12/36 (33.3%) cases (Fig. 1a, Table 1). Grade III staining (>51% positive MDM4 cells) was only observed in one de-differentiated liposarcoma (Tumour SE74). Interestingly, well/de-differentiated liposarcomas and myxofibrosarcomas exhibited significantly higher levels of MDM4 protein expression compared to the rest of the sarcoma cohort (*p* < *0.0001*) (Fig. 1b).

Recently, Wynendaele and colleagues reported that presence of a single nucleotide polymorphism (SNP34091, rs4245739) located 32 nucleotides downstream of the stop codon in the 3’UTR of *MDM4* was associated with statistically significant increased MDM4 protein expression in high-grade ovarian carcinomas^20^. This A>C transversion was reported to create a putative illegitimate target site for *hsa-miR-191*, which only recognised the 3’UTR of the *MDM4-C* allele resulting in decreased *MDM4* mRNA and protein expression. To determine whether SNP34091 regulates MDM4 protein expression in sarcomas, the 3’UTR of *MDM4* was sequenced. Genotypes were as follows, 17 (47.2%) were homozygous for the wild-type allele (A/A), 12 (33.3%) were heterozygous (A/C) and 7 (19.4%) were homozygous for SNP34091 (C/C) (Table 1). Presence of the C allele was not significantly associated with decreased MDM4 protein expression within our sarcoma cohort (AA vs AC: *P* = *0.521*) (AA vs CC: *P* = *0.624*) (Fig. 1c).

### XI-006 induces potent apoptosis in Ewing sarcoma cell lines

**Figure 2:**
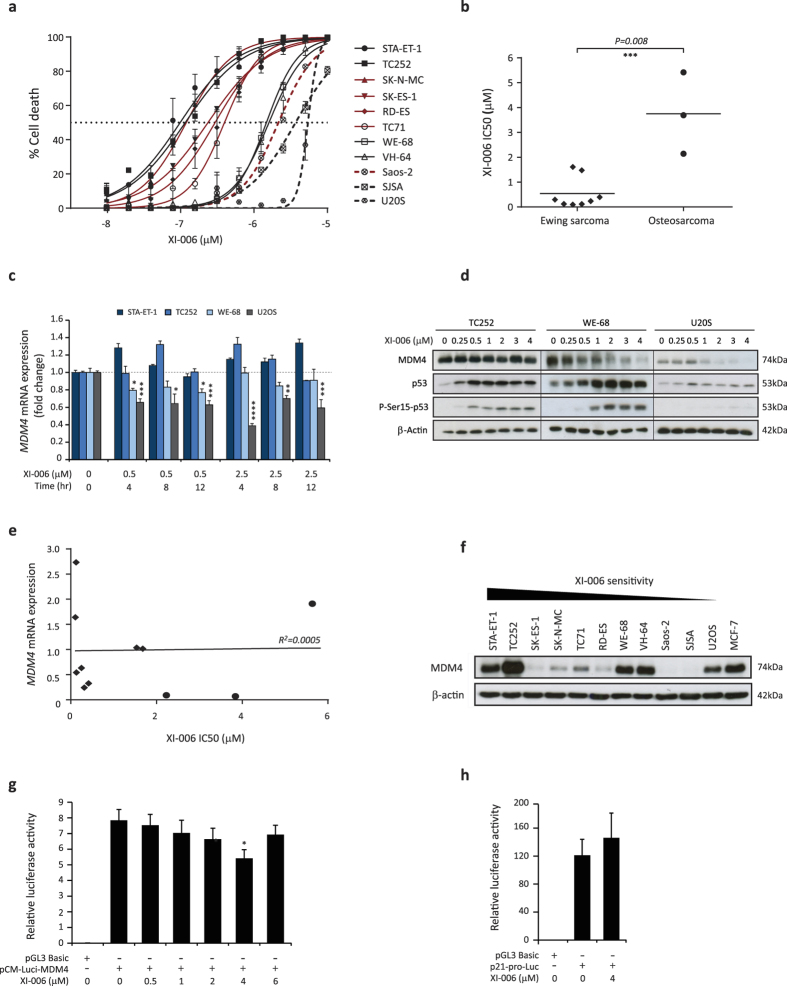
*MDM4* mRNA and protein levels do not confer XI-006 sensitivity (a) XI-006 apoptotic dose response curves of all sarcoma cell lines following 48 hrs of XI-006 treatment. Dashed and red lines denote osteosarcoma and mutant *TP53*/null cell lines respectively. Data represents mean ± STDEV from two independent experiments, duplicate reactions. (b) Correlation between XI-006 apoptotic 48 hr IC50 values determined from 7AAD staining and sarcoma pathology. (c) STA-ET-1, TC252, WE-68 and U20S cells were treated with XI-006 (0, 2.5 and 5 μM) for the indicated times with mRNA expression levels of *MDM4* determined through real-time qPCR analysis. Data represents mean expression (fold change) ± SE from triplicate reactions. (d) TC252, WE-68 and U20S cell lines were treated for 4 hrs with the indicated concentrations of XI-006. MDM4, p53, and phosphorylated p53 (serine 15) protein levels were detected through western blot analysis. β−Actin was used a loading control. (e) Lack of correlation between XI-006 apoptotic 48 hr IC50 values and basal MDM4 mRNA expression levels. ♦• Denotes Ewing sarcoma and osteosarcoma cell lines respectively. (f) Western blot analysis of basal MDM4 protein levels of the cell line cohort. Cell lines ranked in order of XI-006 sensitivity. MCF-7 (MDM4 amplified breast cancer cell line) was used as a positive control. Luciferase assay of (g) MDM4 and (h) p21 promoter activity from U20S cells treated with XI-006 for 6 hrs. Data represents mean ± SE from 3 independent experiments. Asterisk denotes statistical significance (*P < 0.05, **P < 0.01, ***P < 0.001, ****P < 0.0001).

As amplification of *MDM4* is frequently observed in Ewing and osteosarcomas^8^, the anti-tumour activity of XI-006 was evaluated in a panel of eleven Ewing and osteosarcoma cell lines. Sarcoma cell lines were exposed to escalating concentrations of XI-006 (0–10 μM), with the degree of apoptosis determined after 24 and 48 hrs of treatment through 7AAD staining and flow cytometry. A pronounced reduction in cell viability was observed specifically in Ewing sarcoma cell lines at low micro-molar concentrations (Table 2, Fig. 2a). Following treatment, concentrations of XI-006 required to induce 50% apoptosis (IC50) in Ewing sarcoma cell lines ranged from 0.376–2.46 μM and 0.099–1.61 μM, 24 and 48 hr treatment respectively. In contrast, osteosarcoma cell lines required significantly higher levels of XI-006 (*P* = *0.008*) to achieve an IC50, range of 3.60–>10 μM and 2.14–5.41 μM, 24 and 48 hr treatment respectively (Fig. 2b). Importantly, the viability of normal human fibroblasts (IMR90) remained unaffected at these low micro-molar concentrations, IC50 of 8.35 μM and 6.80 μM (24 and 48 hr treatment respectively) (Table 2). Colony formation assays were performed to investigate the long term effect of XI-006 on cellular proliferation. Following 10 days of XI-006 treatment, no colonies were observed at any XI-006 concentration exceeding 0.03 μM in TC252 and RD-ES Ewing sarcoma cell lines, and 0.11 μM in U20S and SJSA osteosarcoma cell lines (Supplementary Fig. S1a,b). In contrast, numerous colonies were observed at the maximum XI-006 concentration tested (3 μM) in IMR90 fibroblasts (Supplementary Fig. S1c), highlighting the tumour specific effect of XI-006.

To examine the cellular outcome following XI-006 treatment, cell lines were treated with XI-006 for 48 hrs, with cell cycle distribution determined through propidium iodide (PI) staining (Supplementary Fig. S2). In the Ewing sarcoma cell lines TC252 and RD-ES, XI-006 induced a dose-dependent decrease in the number of G1-phase cells, and accumulation of cells in SubG1. In contrast, XI-006 effectively arrested cell cycle progression in osteosarcoma cell lines (U20S, Saos-2), depleting the G1 compartment to 18.6–19.9% and increasing the G2 compartment to 45.7–61.5% (3 μM treatment).

### MDM4 mRNA and protein levels do not confer sensitivity to XI-006

As XI-006 was shown to decrease MDM4 expression in breast cancer cell lines^18^, we investigated the effects of XI-006 on both *MDM4* mRNA and protein levels in cell lines with varying *MDM4* genomic^21^, mRNA and protein expression levels (Table 2, Fig. 2f). A maximum 61.2% reduction in *MDM4* mRNA expression was observed in U20S cells (2.5 μM, 4 hrs) following XI-006 treatment; however U20S cells were the least sensitive cell line to XI-006 (Fig. 2c). Although XI-006 did not attenuate *MDM4* mRNA levels in the sensitive Ewing sarcoma cell line TC252, a 23.1% reduction was observed in WE-68 cells (0.5 μM, 12 hrs), the least sensitive Ewing sarcoma cell line. Consistent with these findings, a dose-dependent reduction in MDM4 protein levels following XI-006 treatment was only observed in U20S and WE-68 cells, but not TC252 cells (Fig. 2d). We next determined whether basal *MDM4* mRNA and protein levels in our cohort of cell lines confers sensitivity to XI-006. Unexpectedly, no correlation between *MDM4* mRNA or protein levels and XI-006 sensitivity was observed (R^2^ = 0.0005) (Table 2, Fig. 2e,f). To confirm whether XI-006 can repress MDM4 promoter activity, luciferase assays of U20S cells treated with escalating concentrations of XI-006 for 6 hrs were performed. A significant decrease in MDM4 promoter activity was only observed at 4 μM (*P* = *0.05*), equating to a 30.5% reduction in activity (Fig. 2g). No effect on p21 promoter activity was observed at this dose (*P* = *0.491*) (Fig. 2h). As X1-006 had no effect on MDM4 promoter activity or mRNA/protein levels at concentrations required to induce 50% apoptosis in the most sensitive Ewing sarcoma cell lines (<0.5 μM), this suggest that the ability of XI-006 to impart apoptosis occurs independently of MDM4.

### XI-006 cytotoxicity occurs independently of TP53

We next examined whether XI-006 can induce expression of *TP53* target genes implicated in apoptosis (*BAX*, *PUMA*), cell cycle arrest (*CDKN1A*) and p53 regulation (*MDM2*). Wild-type *TP53* Ewing (TC252, STA-ET-1, WE-68) and osteosarcoma (U20S, SJSA) cell lines were treated with XI-006 (0.5, 2.5 μM), with target gene expression assessed through real-time qPCR assays (Supplementary Fig. S3a). XI-006 dramatically increased mRNA expression levels of all *TP53* target genes in Ewing sarcoma cell lines in a dose and time dependent manner. A maximum 63 fold increase in *BBC3* levels was observed in STA-ET-1 cells (2.5 μM, 4 hrs). In contrast, induction of these target genes was significantly lower in all osteosarcoma cell lines, in particular *BAX* and *CDKN1A*, where no induction was observed for all time points and XI-006 concentrations.

To determine whether induction of p53 target genes specifically in Ewing sarcoma cells could be attributed to activation and stabilisation of p53, p53 protein levels following XI-006 treatment were examined. Activation and stabilisation of p53 protein levels was only observed in TC252 and WE-68 Ewing sarcoma cell lines but not U20S cells following 4 hrs of XI-006 treatment (Fig. 2d). Furthermore, phosphorylation of p53 at serine 15 which is synonymous with ATM dependent activation of the DNA damage pathway^22^ was observed at XI-006 concentrations exceeding 0.5 μM. Although XI-006 induced expression of *TP53* target genes, the cytotoxic effects of XI-006 were shown to be *TP53* independent, as XI-006 sensitivity was not correlated with *TP53* status (*P* = *0.190*) (Supplementary Fig. S3b). Indeed, the least sensitive Ewing (WE-68, VH-64) and osteosarcoma (U20S, SJSA) cell lines harboured wild-type p53 (Table 2). To further examine the role of *TP53* in XI-006 cytotoxicity, *TP53* wild-type and null HCT116 isogenic cell lines were treated with XI-006 (0–10 μM) (Supplementary Fig. S3c). No significant difference in both XI-006 IC50 values (24 hr: *P* = *0.230*, 48 hr: *P* = *0.505*) or relative viability at any concentration tested was observed, endorsing the p53-independent cytotoxic effects of XI-006.

### Low micro-molar concentrations of XI-006 do not induce DNA damage

**Figure 3:**
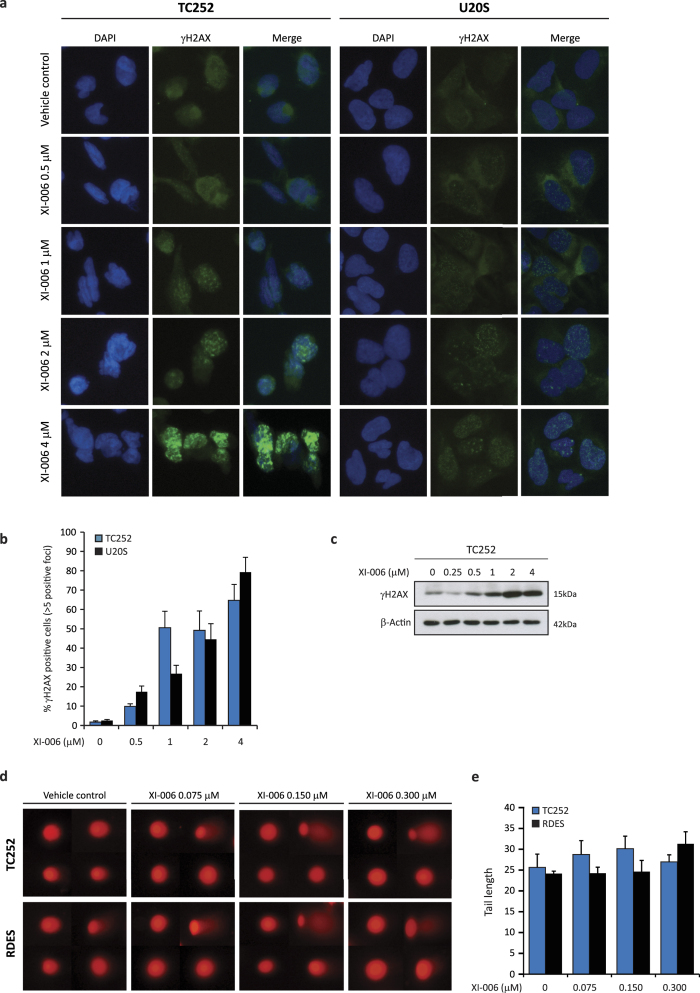
XI-006 does not induce double-strand break DNA damage at low micro-molar concentrations (a) Representative images of γH2AX foci formation (immunofluorescence) from TC252 and U20S cells treated with the indicated concentrations of XI-006 for 4 hrs. Cells were stained with DAPI (blue) and γH2AX (green). (b) Percent nuclei positive for γH2AX foci (>5) from cells treated as in (a) (mean ± STDEV from duplicate wells). (c) Western blot analysis of γH2AX protein levels in TC252 cells treated as in (a). β-Actin was used as the loading control. (d) Representative images of neutral comet assays from TC252 and RDES cells treated with low dose XI-006 (0.075, 0.150, 0.300 μM) for 20 hrs. (e) Quantification of tail length from cells treated as in (d), mean ± SE.

Previous reports proposed that XI-006 activates the DNA damage response pathway leading to a delay in cell cycle progression^23^. As XI-006 cytotoxicity occurs independently of MDM4, we sought to address whether XI-006 drives apoptosis through DNA damage. Clustering of phosphorylated H2AX moieties (γH2AX foci) at the site of double-strand breaks (DSBs) is one of the earliest events indicative of DNA damage^24^. Indeed a dose dependent increase in γH2AX foci was detected through immunofluorescence analysis following 4 hrs of XI-006 treatment in TC252 and U20S cells (Fig. 3a). At 4 μM 64.7–79.3% of cells (TC252 and U20S respectively) displayed >5 positive γH2AX foci. However at low doses required to induce apoptosis in the most sensitive Ewing sarcoma cell lines (0.5 μM), positive γH2AX foci were only detected in 9.9–17.4% of cells (Fig. 3b). In agreement with these findings XI-006 induced γH2AX foci formation was correlated with H2AX phosphorylation (Fig. 3c). Neutral comet assays were also employed to determine whether low dose XI-006 (0.075, 0.150, 0.300 μM) induces DSB’s following long term exposure (20 hrs) n TC252 and RD-ES cells (Fig. 3d). No significant difference in comet tail length was observed in comparison to vehicle control treated cells at any XI-006 concentration tested (Fig. 3e).

The serine/threonine protein kinase ATM (*ataxia telangiectasia mutated*) is critical for sensing and co-ordinating repair of DNA DSBs. To further confirm that the cytotoxic effects of XI-006 was not due to DNA damage at low concentrations, Ewing cells (TC252, RD-ES and WE-68) were pre-treated with the ATM inhibitor KU-55933, before the addition of XI-006 (0.02–5 μM). A significant reduction in both *ATM* and *ATR* mRNA expression (82.0% and 87.5% respectively) was observed following monotherapy KU-55933 treatment (5 μM) (Supplementary Fig. S4a,b). No significant difference in XI-006 apoptotic IC50 values (24 and 48 hr treatment) was observed following ATM inhibition across all cell lines tested (Supplementary Fig. S4c).

Previous chemogenomic profiling studies suggested that XI-006 imparts its cytotoxic effect through the activation of the DNA-damage-response pathway. Phosphorylation of the N-terminal domain of *EWS-FLI1*, the hallmark gene fusion of Ewing sarcoma, at Thr^25^ has been reported in response to mitogen or DNA alkylating agent induced DNA damage^26^. This post-translational modification was found to be catalysed by p38α/p38β mitogen-activated protein kinases (MAPKs). As all Ewing sarcoma cell lines utilised in this study harbour the *EWS-FLI1* fusion, we examined the role p38α/p38β MAPKs in XI-006 sensitivity. TC252 and RD-ES cells were pre-treated with the p38α/p38β MAPK inhibitor BIRB 796 (0.1, 1 and 10 μM) before the addition of XI-006 (0–2.5 μM) for 24 and 48 hrs. BIRB 796 was previously shown to inhibit p38α/p38β MAPKs specifically at 0.1 μM, p38y/p38δ MAPKs at 1 μM and completely suppress the activation and activity of all JNKs at 10 μM^26^ in Ewing cell lines. Treatment with BIRB 796 had no effect on XI-006 induced cytotoxicity (24 and 48 hrs), as a significant reduction in apoptosis (>30%) was not observed at any BIRB 796 concentration (Supplementary Fig. S5).

**Figure 4:**
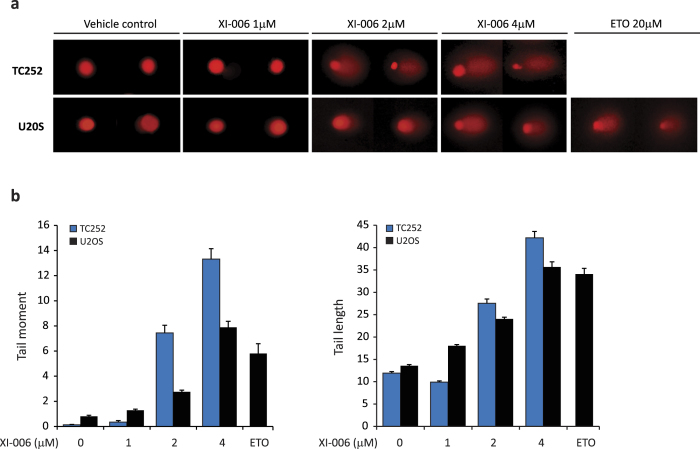
XI-006 does not induce single-strand break DNA damage at low micro-molar concentrations a) Representative images of alkaline comet assays from TC252 and U20S cells treated with XI-006 (1, 2, 4 μM) for 4 hrs. Etoposide (ETO) was used as a positive control (b) Quantification of tail length and tail moment from cells treated as in (a) mean ± SE.

Finally, alkaline comet assays were employed to investigate whether XI-006 induces single-strand break DNA damage. Following 4 hrs of XI-006 treatment, a significant increase in comet tail and length was only observed at concentrations exceeding 2 μM (Fig. 4). Collectively these results suggest that XI-006 concentrations (<0.5 μM) required to induce apoptosis in sensitive Ewing sarcoma cells lines cannot be attributed to a DNA damage mechanism of action

### XI-006 synergises with olaparib, actinomycin D, doxorubicin and etoposide

As conventional single-agent cancer therapy increases the likelihood of the emergence of resistant cancer cell clones, combination therapies are required to achieve maximal therapeutic response. As such, the ability of XI-006 to enhance the cytotoxic effects of four standard Ewing sarcoma chemotherapeutic agents (vincristine, actinomycin D, doxorubicin or etoposide) was assessed in five Ewing sarcoma cell lines (TC252, STA-ET-1, WE-68, RD-ES, and SK-N-MC). Modest synergistic combination indexes (CI < 1) were observed in 4/5 cell lines for actinomycin and doxorubicin and 3/5 cell lines tested for etoposide (CI range 0.753–0.989) over multiple chemotherapeutic doses (Supplementary Table S2, Supplementary Fig. S6). Although antagonistic CI values were obtained between XI-006 and vincristine (inhibitor of microtubule assembly), a strong correlation (R^2^ = 0.722) was observed between XI-006 and vincristine IC50 values in all Ewing sarcoma cell lines, suggesting that these two agents may have a similar mechanism of action (Supplementary Fig. S7).

**Figure 5:**
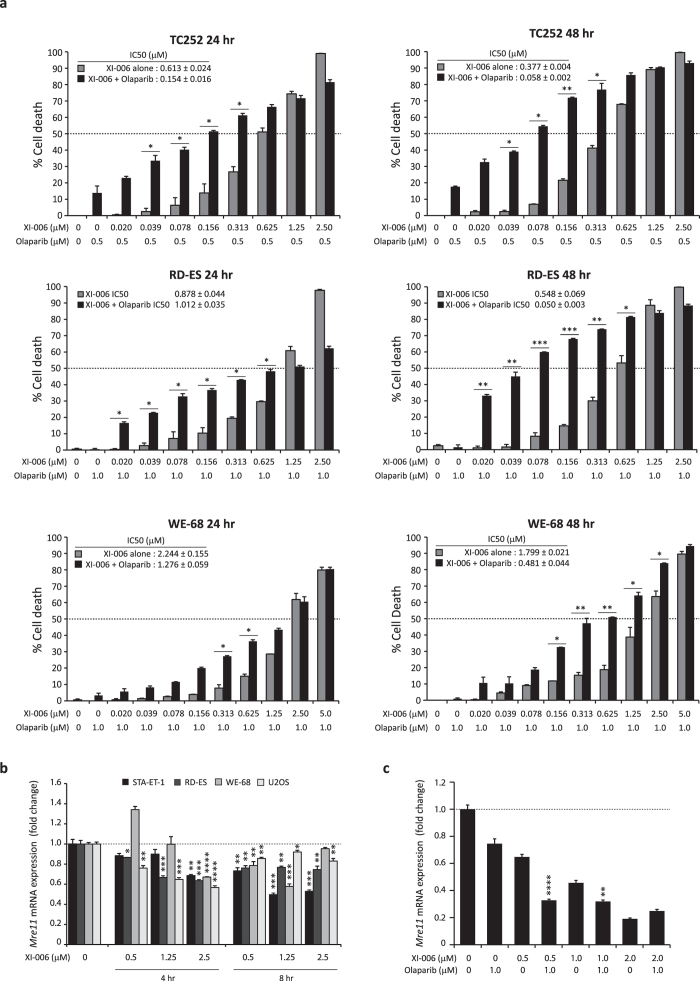
Inhibition of PARP potentiates the cytotoxic effects of XI-006 a) TC252, RD-ES and WE-68 were pre-treated with olaparib (0.5 or 1 μM) or vehicle control (DMSO) for 2 hrs, prior to the addition of XI-006 (0–5 μM). Cell viability was determined through 7AAD staining (24 and 48 hrs post XI-006 treatment) and analysed by flow cytometry. Data represents average percentage cell death ± STDEV from duplicate reactions. Asterisk denotes significant increase in apoptosis compared to XI-006 alone (*15–30%, **31–45%, ***>46% increase). (b) STA-ET-1, RDES, WE-68 and U20S cells were treated with XI-006 (0.5, 1.25, 2.5 μM) for 4 and 8 hrs. mRNA expression levels of *Mre11* were determined through real-time qPCR analysis. Data represents mean expression (fold change) ± SE from triplicate reactions. (c) RD-ES cells were pre-treated with Olaparib for 2 hrs prior to the addition of XI-006 (0.5, 1, 2 μM) for an additional 4 hrs. *Mre11* mRNA expression levels determined as in (b). Asterisk denotes statistical significant reduction in expression compared to either vehicle control (b) or XI-006 treatment alone (c) (*P < 0.05, **P < 0.01, ***P < 0.001, ****P < 0.0001).

The EWS-FLI fusion protein has been shown to drive expression of PARP1 (*poly-ADP-ribose polymerase*), which subsequently further promotes transcriptional activation by *EWS-FLI*^27^. The primary function of PARP is to sense and mediate repair of DNA single strand breaks (SSB). Therefore we determined if XI-006 can synergise with olaparib (PARP inhibitor) in *EWS-FLI* positive Ewing sarcoma cell lines. TC252, RD-ES and WE-68 cells were pre-treated with olaparib prior to addition of XI-006 (0–5 μM). Potent synergy between these two agents was observed in all cell lines tested and across multiple XI-006 concentrations, 24 and 48 hrs post XI-006 treatment. A maximum 51.9% increase in apoptosis was observed in RD-ES cells when these two agents were combined (48 hr XI-006 treatment) (Fig. 5a). Olaparib did not abrogate the cytotoxic effects of XI-006 at any concentration tested, suggesting that XI-006 does not induce DNA damage via single stranded breaks at low doses.

In addition to repair of SSB, PARP detects stalled replication forks and attracts Mre11 (*meiotic recombination 11*) for end processing to facilitate replication restart and recombination repair^28^. As XI-006 was previously shown to induce a significant delay in replication^23^, *Mre11* mRNA expression levels following XI-006 treatment was assessed. A decrease in *Mre11* mRNA expression was observed across all cell lines tested, with a maximum 50.1% reduction observed in STA-ET-1 cells (8 hr treatment) (Fig. 5b, Supplementary Fig. S8). As PARP mediates the recruitment Mre11 to stalled replication forks, *Mre11* expression in RD-ES pre-treated with olaparib (1 μM) before the addition of XI-006 was assessed. Olaparib treatment alone resulted in a significant 25% decrease in *Mre11* mRNA expression (Fig. 5c). Co-treatment with XI-006 (0.5 and 1 μM) further repressed *Mre11* mRNA expression levels, maximum 50.4% reduction compared to XI-006 treatment alone observed. Together, these findings indicate that in the absence of DNA damage low micro-molar concentrations of XI-006 can potentiate the cytotoxic effects of olaparib due to down-regulation of *Mre11* expression.

### HEG1, FLOT1, UTRN and EDIL3 are differentially expressed in Ewing and osteosarcoma cells following XI-006 treatment

mRNA sequencing of XI-006 treated and untreated cell lines was employed to identify genes responsible for XI-006 cytotoxicity. Across all Ewing and osteosarcoma cell lines eleven genes were found to be either significantly (*P* < *0.05*, Bonferroni correction) repressed (*KIF20A*, *IDH1*, *SCD*, *GPSM2*, *EIF2AK4*, *HIBCH*) or induced (*STK19*, *DNAJC24*, *MTG2*, *FAM175B*, *CYB5D1*) following non DNA-damaging XI-006 treatment (0.5 μM) (Table 3). Real-time qPCR analyses confirmed that *KIF20A* which is required for normal cleavage furrow ingression and cytokinesis during cell division^29^ and *IDH1* (cytosolic NADP dependent enzyme) were repressed on average by 51.2% and 41.3% respectively across all cell lines following XI-006 treatment (Supplementary Fig. S9).

We next sought to identify genes that were differentially expressed between Ewing and osteosarcoma cell lines following XI-006 treatment. Four genes (*UTRN*, *HEG1*, *FLOT1*, *EDIL3*) were identified and validated through real-time qPCR analysis (Table 3, Supplementary Fig. S10). Of particular interest *UTRN* which mediates several mitochondria dependent apoptosis pathways, was found to be significantly upregulated in Ewing cell lines (average 22.7% increase) which undergo apoptosis following XI-006 treatment, and repressed in osteosarcoma cell lines (average 31.9% decrease) (*P* = *0.025*) which undergo cell-cycle arrest following XI-006 treatment.

## Discussion

Despite the use of aggressive multi-modal therapeutic strategies, five year survival rates for relapsed Ewing sarcoma patients is <30% and in such cases no standard therapy currently exists for second line treatment. Despite the emerging role of MDM4 (structural homologue of MDM2) in the pathogenesis, maintenance, and chemo-resistance of human cancer, there are currently no selective MDM4 antagonists undergoing clinical trial evaluation. As MDM4 gene amplification is a characteristic of both Ewing and osteosarcoma^8^, this study assessed the therapeutic potential of XI-006, a small molecule thought to attenuate MDM4 promoter activity, for the treatment of sarcoma. Indeed, MDM4 IHC analysis of our sarcoma cohort detected MDM4 protein expression (>10 positive cells) in 33.3% (12/36) of cases and was highly prevalent in well/de-differentiated liposarcomas and myxofibrosarcomas (Fig. 1).

Low micro-molar concentrations of XI-006 induced rapid apoptosis specifically in Ewing sarcoma cell lines (IC50 0.099–1.61 μM) in the absence of both observable DNA damage and effect on MDM4 expression levels (Table 1, Fig. 2). Cell line sensitivity to XI-006 was not correlated with *MDM4* mRNA or protein levels, and reduction of *MDM4* mRNA and protein levels were only observed in the least sensitive Ewing sarcoma and osteosarcoma cell lines at high XI-006 concentrations (>1 μM) that also induced DNA damage (Figs 3 and 4). It is known that DNA damage induces ATM/Chk2 dependent phosphorylation of several MDM4 C-terminal residues (S342, S367, S403), resulting in degradation of MDM4 and activation of p53^25^. Indeed, ATM mediated phosphorylation of serine 15 in p53 was observed in Ewing sarcoma cell lines following treatment with double-strand break inducing concentrations of XI-006 (>0.5 μM). As only a maximum 30.5% reduction in MDM4 promoter activity was observed following 4 μM XI-006 treatment (Fig. 2), our findings suggest that XI-006 cytotoxicity in Ewing sarcoma cell lines cannot be attributed to repression of MDM4 activity.

The introduction of systemic chemotherapy in the 1960’s greatly improved survival rates for patients with localised Ewing sarcoma^30^, hence it is imperative that XI-006 can synergise with current chemotherapeutic protocols as well as novel agents. In addition to synergising with doxorubicin, etoposide and actinomycin D (CI range 0.753–0.989), a maximum 51.9% increase in apoptosis was observed when XI-006 was combined with the PARP inhibitor olaparib (Fig. 5).

PARP-1 is a member of the base excision repair pathway that sensors and modulates the spatial and temporal organization of single-strand break repair^31^. Inhibition of PARP-1, results in the accumulation of persistent single-strand breaks which are converted to lethal double-strand breaks upon replication. Since the 1990’s it has been known that Ewing sarcomas express high levels of PARP-1^32^, and the premise of PARP inhibition as a therapeutic avenue for the treatment of Ewing sarcoma has been furthered strengthened by several key studies. Firstly, large-scale drug screening (130 compounds) in >600 human cancer cell lines identified a highly significant association between *EWS-FLI1*, the hallmark translocation of Ewing sarcoma, and sensitivity to the olaparib. Indeed, *FLI1* expression levels in Ewing sarcoma cell lines were highly correlated with olaparib sensitivity^33^. This study was complemented by Brenner and colleagues, who demonstrated that the *EWS-FLI1* fusion acted in a positive feedback loop to maintain the expression of PARP1 which was required for *EWS-FLI*–mediated transcription^27^. Furthermore, DNA damage induced by expression of *EWS-FLI1* was potentiated by PARP1 inhibition *in vitro*. As PARP1 inhibitors have exhibited promising activity in early clinical trials^34^, phase I/II trials of PARP inhibitors (olaparib and BMN-673) are currently undergoing investigations in adults with recurrent and metastatic Ewing sarcoma (NCT01583543), and patients with locally advanced or metastatic solid tumours (NCT02049593).

Previous chemogenomic profiling studies suggested that XI-006 (referred to as NSC-207895) activates the DNA-damage-response pathway through an indirect mechanism leading to a significant delay in replication and cell cycle progression^23^. Replication stress, defined as the slowing or stalling of replication fork progression and/or DNA synthesis, has severe implications for genome stability and cell survival^35^. Several studies have implicated that PARP binds to and is activated at stalled replication forks that contain small gaps (<4 nucleotides) or short ssDNA regions, and mediates the recruitment of Mre11^28^. Mre11 is a key component of the MRN (Mre11-Rad50-Nbs1) complex, which is vital for double-strand break (DSB) recognition, replication fork stabilization, ATM/ATR activation and the initiation of end resection required for replication restart and homologous recombination (HR)^36^. Inhibition or loss of PARP impairs Mre11 localisation to stalled forks, RPA and RAD51 foci formation, HR and replication restart. Indeed numerous studies have shown that due to impaired HR DNA repair, loss of Mre11 expression sensitizes breast^37^, colorectal^38^,^39^, endometrial^40^, and haematological cancers^41^ to PARP-inhibitors. In the absence of DNA damage, our findings demonstrate that low dose XI-006 rapidly down regulates the expression of Mre11 (Fig. 5) and this repression in further enhanced in the presence of olaparib (Fig. 5), resulting in potent apoptosis. As such, these findings provide a strong rationale for further investigations into the combinatorial approach of PARP inhibitors with XI-006.

To elucidate the genes specifically responsible for Ewing sarcoma apoptotic XI-006 sensitivity, mRNA sequencing was employed and identified four genes significantly differentially expressed between Ewing and osteosarcoma cell lines following XI-006 treatment (Table 3). Of particular relevance, *UTRN* (Utrophin) was significantly upregulated in Ewing sarcoma cell lines but repressed in osteosarcoma cell lines (*P* = *0.025*). The GTPase *UTRN* also known as *Drip1*, mediates outer mitochondrial membrane fission and is essential for the normal progression of several mitochondria dependent apoptosis pathways^42^. Upon induction of apoptosis, *UTRN* is recruited from the cytosol to the mitochondrial outer membrane, where it colocalizes with Bax at fission sites and mediates the release of apoptotic regulatory proteins including cytochrome *c* prior to caspase activation^43^,^44^. The expression of *FLOT1* (flotillin) was also significantly repressed in osteosarcoma cell lines (*P* = *0.044*) compared to Ewing. The flotillin family of proteins have been implicated in numerous cellular processes such as actin-cytoskeleton reorganization, endocytosis, adhesion and transduction of cellular signals^45^. Knockdown of *FLOT1* has been shown to significantly impair cell proliferation and tumourigenicity of breast and esophageal squamous cell carcinoma cells *in vitro* and *in vivo* through the Akt/FOXO3a pathways^46^,^47^. Indeed, silencing of *FLOT1* induced G1-S-phase arrest of breast cancer cells due to upregulated expression of the CDK inhibitors *p21*^*Cip1*^ and *p27*^*Kip1*^. Following XI-006 treatment, both *UTRN* and *FLOT1* were strongly repressed in osteosarcoma cell lines (31.9% and 33.8% decrease in mRNA expression respectively) but not in Ewing cell lines. This may be the basis of the finding that XI-006 induces apoptosis in Ewing sarcoma cell lines but cell cycle arrest in osteosarcoma cell lines (Supplementary Fig. S2).

Expression profiling also revealed that following XI-006 treatment, eleven genes were globally repressed or induced across all Ewing and osteosarcoma cell lines. Three of these genes which were downregulated, *KIF20A*, *IDH1* and *GPSM2* (44.49%, 37.14% and 33.34% reduction respectively), have been implicated in cell proliferation. KIF20A belongs to the family of kinesin microtubule-dependent motor proteins, which are required for bipolar spindle assembly, chromosome alignment, chromosome segregation, and cytokinesis^48^. Similarly GPSM2 is required for spindle cell orientation towards the interphase long-axis^49^. Several studies have demonstrated the essential role of KIF20A in cytokinesis and maintenance of cell viability. Microinjection of anti-KIF20A antibody was shown to induce multi-nucleation in Hela cells^50^ and knockdown of endogenous KIF20A expression markedly attenuated the growth of pancreatic and gastric cancer cells^51^,^52^. As sensitivity to XI-006 was strongly correlated with vincristine sensitivity (R^2^ = 0.722) (Supplementary Fig. S7), a widely used chemotherapeutic that inhibits microtubule assembly and induces tubulin self-association into coiled spiral aggregates^53^, this supports that the mechanism of action of XI-006 at low, micro-molar concentrations (<0.5 μM) can be attributed to inhibition of cell division and cycle regulators and not DNA damage.

In summary, our findings demonstrate that XI-006 is a promising new potential therapeutic for the treatment of Ewing sarcoma as it induced potent p53-independent apoptosis at non-DNA damaging concentrations specifically in Ewing sarcoma cell lines. Notably, strong synergy was observed with olaparib, a PARP inhibitor that is gaining significant interest for the treatment of solid cancers. As such, our preclinical findings warrant further pharmacokinetic and pharmacodynamic investigations of XI-006 *in vivo*.

## Methods

### Cell lines and reagents

Ewing sarcoma cell lines were cultured as previously described^21^ and supplied by G. Hamilton (University of Vienna, Austria) (TC252, TC71), F. van Valen (Westfälische-Wilhelms-University, Germany) (WE-68, VH-64), P. Ambros (St. Anna Children’s Hospital, Austria) (STA-ET-1) and V. Russo (Murdoch Children’s Research Institute, Australia) (SK-N-MC). SK-ES-1, RD-ES cell lines were purchased from American Type Tissue Culture. Osteosarcoma cells (SJSA, U20S and Soas-2) were supplied by A. Evdokiou (University of Adelaide, Australia).

XI-006 and BIRB 796 were purchased from Merck Millipore, KU-55933 and olaparib (AZD2281) were purchased from Selleck. Vincristine sulfate (Hospira), doxorubicin HCI (Pfizer), actinomycin D/Cosmegen (Lundbeck) and etoposide (Royal Adelaide Hospital, Australia) were supplied by M.P Brown (Centre for Cancer Biology, Australia).

### Sarcoma tissue cohort

Chemotherapy/radiotherapy naïve tumour specimens were collected from thirty-six patients with sarcoma (22 males, 14 females) undergoing surgical resection/core biopsy at three clinical institutions; Royal Adelaide Hospital (RAH), Calvary Wakefield Hospital, and St Andrew’s Hospital between 2010 and 2013. Patient consent was obtained for accrual of surgically excised tissue. Study was approved by the Royal Adelaide Hospital Human Ethics Committee (RAH Protocol #100505). The different morphological subtypes were represented by nine undifferentiated pleormorphic sarcomas, eight liposarcomas (four well-differentiated, two de-differentiated, one pleomorphic and myxoid), six leiomyosarcomas, five myxofibrosarcomas, two osteosarcomas, two synovial and one Ewing sarcoma, angiosarcoma, chondrosarcoma and radiation induced sarcoma. Twenty patients were previously described^19^. All methods were carried out in accordance with *National Health and Medical Research* (NHMRC) approved guidelines.

### Immunohistochemistry

MDM4 immunohistochemical protocol was adapted from^54^. Briefly, FFPE (4 μm thickness) were deparaffinised by serial immersion in a xylene-to-ethanol solvent gradient. After citrate buffer (0.001 mol/L, pH 6.0) antigen retrieval, slides were quenched in 3% hydrogen peroxide for 5 mins to eliminate endogenous peroxidase activity. Sections were blocked with normal goat serum (30 mins) and immuno-labelled with rabbit HdmX/MDM4 (1:250, IHC-00108, Bethyl Laboratories) overnight at 4 °C. Digital images were acquired using a Nanozoomer Digital Pathology Scanner, at x40 magnification. To determine the percentage of positive MDM4 cells, a minimum of 80 cells per field of view (four) were assessed.

### MDM4 SNP34091 genotype analysis

DNA from sarcoma tissues was isolated using the DNeasy Blood and Tissue Kit (Qiagen) according to the manufacturer’s instructions. The 3’UTR region of MDM4 was amplified using the following primer pair^55^ forward: 5′ACGGGCCATCTTGTCACTTGTT 3′ and reverse: 5′ACCTGACTGCT GCATAAAGTAATCCAT 3′, to amplify a 355 base pair (bp) product. PCR was performed using 100 ng of genomic DNA and FastStart Taq DNA Polymerase (Roche) using the following parameters, enzyme activation 95 °C 3 mins, followed by 45 cycles of denaturation at 95 °C 30 secs, annealing at 57 °C 15 secs, and extension at 72 °C 60 secs, followed by 1 cycle of final extension at 72 °C 10 mins. Reactions were processed on an ABI Hitachi 3730 DNA analyser.

### Apoptosis and Cell cycle analysis

For viability assays, cell were seeded in 96-well micro-titer plates at a density of 3 × 10^4^ cells/well and treated with XI-006 alone or in combination with chemotherapeutics agents. For inhibitor studies, cells were pre-treated with olaparib, KU-55933 or BIRB 796 for 2 hrs prior to the addition of XI-006. Following treatment for 24 and/or 48 hrs, cells were centrifuged at 2500 rpm for 5 mins, washed in phosphate buffered saline (PBS) and stained with 7-amino-actinomycin-D solution (7AAD, 2 mg/mL, Invitrogen) for 10 mins at room temperature.

For cell cycle analysis, XI-006 and vehicle control treated cells were permeabilized with cold 70% ethanol overnight, and stained with a solution containing 50 μg/ml propidium iodide (PI, Sigma Aldrich), 0.05% Triton-X and 100 μg/ml RNase A at 37 °C for 40 mins. Cell viability and DNA content was determined through the use of a FACS Calibur flow cytometer (Becton-Dickinson Immunocytometry Systems) with cell cycle profiles and viability analyzed using FLOWJO software (V7.6.5).

### Luciferase Assay

U20S cells (3.2 × 10^4^) were seeded overnight in 24-well plates (triplicate wells per treatment) and transfected with 200 ng of pCM-luci-MDM4 or p21-pro-Luc reporter constructs and 25 ng of pRL-TK plasmid (Promega) using Lipofectamine LTX (Invitrogen) according to the manufacturers’ instructions. pCM-Luci-MDM4 was kindly supplied by C. Yan (GRU Cancer Center). Cells were treated with vehicle control or XI-006 for 6 hrs with Dual-luciferase reporter assays (Promega) performed according to the manufacturer’s instructions.

### Real-time qPCR analysis

Total RNA was extracted using RNeasy mini kit (Qiagen), using on-column RNase-free DNase digestion according to the manufacturer’s instructions. cDNA was synthesized by reverse transcribing 600 ng of total RNA using random primers (Promega) and Moloney murine leukemia virus reverse transcriptase (H^_^; Promega). Real-time qPCR reactions were performed using iTaq Universal Sybr Green Supermix (BIORAD) and processed on a CFX Real-Time PCR detection system (BIORAD). Cycling parameters were as follows: 95 °C for 3 mins, followed by 45 cycles of denaturation at 95 °C for 10 secs, annealing at 59–63 °C for 15 secs, and extension at 72 °C for 30 secs. Relative target mRNA expression was determined using the ΔCT method from triplicate reactions, with the levels of gene expression normalized to the relative average Ct value of Peptidylprolyl Isomerase-G (PPIG). Primer sequences and annealing temperatures are listed in Supplementary Table S1.

### Western blot

Western blot analysis was performed as previously described^21^. Whole protein lysates (5–20 μg) were resolved using SDS PAGE electrophoresis, and probed overnight at 4 °C with the following primary antibodies MDM4 (1:500; A300-287A, Bethyl Laboratories), p53 DO1 (1:1000, Santa Cruz Biotechnology), Phospho-p53 (Ser15) (1:500, Cell Signalling), Phospho-Histone H2A.X (Ser139) clone JBW301 (1:500, Millipore) and β-Actin (1:1000, AC-15, Sigma).

### Immunofluorescence

TC252 and U20S cells (1.5 × 10^4^ cells per 6 well chamber) were seeded on microscopes slides and treated with XI-006 (0.5, 1, 2 and 4 μM) or vehicle control (DMSO) for 4 hrs. Cells were fixed with 10% neutral-buffered formalin, washed with PBS and blocked with 5% bovine albumin serum with 0.3% Triton-X 100 (Sigma-Aldrich). Slides were washed in PBS (3 × 5 min), then incubated with 1 μg/ml biotinylated mouse anti-human anti-phospho-histone H2AX (ser139) (JBW301, Millipore) overnight at 4 °C. After further washing, samples were incubated with 5 μg/ml streptavidin-Alexa Fluor 488 (Life Technologies) followed by counterstaining with 1 μg/ml DAPI. Slides were examined using an Olympus IX71 microscope (x40 magnification) with CellSens Standard (v1.6) software. Images were analysed using ImageJ (v1.45) (National Institute of Health).

### Comet Assay

Neutral and alkaline comet assays were performed with the Trevigen CometAssay kit according to the manufacturer’s protocol. Briefly, 3.5 × 10^5^ cells were plated in 6 well plates and treated with XI-006 or vehicle control for 4 or 20 hrs. Lysed cells were subjected to electrophoresis for 40 mins at 30 V (300 mA) at 4 °C. Cells were stained with 2.5 μg/ml PI for 15 mins and visualised with an Olympus IX71 microscope (x20 magnification) with CellSens Standard (v1.6) software. Tail length and moment were assessed using AutoComet software (TriTek) from a minimum of 60 cells.

### mRNA sequencing

Ewing and osteosarcoma cell lines (n = 11) were treated with XI-006 (0.5 μM) or vehicle control for 4 hrs. One μg of RNA was used for polyA selection and library construction with NEBNext UltraT RNA Library Prep Kits for Illumina sequencing, according to the manufacturer’s instructions (E7530 Version 2). The mRNA library size was validated with the Agilent BioAnalyzer on High Sensitivity chips, with yield determined with a Life Technologies Qubit 2.0 Fluorometer. The mRNA libraries were pooled and sequenced across five lanes of an Illumina HiSeq 2500 flowcell (1 × 50 bp reads) at the Australian Cancer Research Foundation Cancer Genomics Facility (Adelaide, Australia). Reads were trimmed for the NEB single end adapter “AGATCGGAAGAGCACACGTCTGAACTCCAG TCAC” with Cutadapt v1.3, requiring a minimum overlap of 5, allowing a 20% error rate and discarding trimmed sequences shorter than 18 bases. Reads were mapped to the UCSC hg19 genome and GTF annotations with Tophat 2.0.9 using default parameters. Gene counts were performed with HTSeq-count v0.6.1p1 using gene_id as the GTF feature ID.

### Statistics

Combination Index (CI) values were used to determine synergy between XI-006 and cytotoxic agents. A CI value of <1, =1 and >1 indicates synergistic, additive and antagonistic effects respectively^56^. *P* values were calculated using Student t test using Graph Pad Prism Version 6.

## Additional Information

**How to cite this article**: Pishas, K. I. *et al*. XI-006 induces potent p53-independent apoptosis in Ewing sarcoma. *Sci. Rep*. **5**, 11465; doi: 10.1038/srep11465 (2015).

## Supplementary Material

Supplementary Information

## Figures and Tables

**Table 1:** Clinical characteristics and MDM4 SNP34091 genotype of the sarcoma cohort.

Sarcoma ID	Patient Gender	Pathology	% Positive MDM4 cells	MDM4 IHC Grade	MDM4 SNP34091
SE74*	M	De-differentiated liposarcoma	51.8 ± 2.3	3	A/A
SE73	M	Well-differentiated liposarcoma	36.2 ± 5.4	2	A/A
SE10*	M	Well-differentiated liposarcoma	30.2 ± 1.8	2	A/C
SE18*	M	Myxofibrosacroma	28.8 ± 2.5	2	C/C
SE31	F	Undifferentiated pleomorphic sarcoma	23.8 ± 5.1	1	A/A
SE13*	F	Myxofibrosacroma	23.6 ± 3.7	1	A/C
SE15*	F	Myxofibrosacroma	18.3 ± 1.7	1	A/A
SE1	M	Myxofibrosacroma	17.7 ± 2.0	1	C/C
SE27*	F	Radiation induced sarcoma	16.5 ± 1.9	1	A/A
SE61*	M	Well-differentiated liposarcoma	15.4 ± 2.6	1	A/A
SE7*	M	Pleomorphic liposarcoma	12.9 ± 2.8	1	A/C
SE24*	M	Leiomyosarcoma	10.7 ± 4.0	1	A/C
SE35*	M	Myxoid liposarcoma	6.1 ± 1.5	0	A/C
SE72*	M	Undifferentiated pleomorphic sarcoma	5.8 ± 2.3	0	A/A
SE39*	M	De-differentiated liposarcoma	4.1 ± 2.3	0	A/A
SE54	M	Well-differentiated liposarcoma	3.5 ± 1.0	0	C/C
SE58*	F	Myxofibrosacroma	3.2 ± 1.4	0	A/A
SE100	M	Chondrosarcoma	2.6 ± 0.6	0	A/C
SE43*	F	Undifferentiated pleomorphic sarcoma	2.5 ± 1.3	0	A/A
SE108	F	Undifferentiated pleomorphic sarcoma	2.4 ± 2.0	0	C/C
SE66	F	Leiomyosarcoma	1.9 ± 1.0	0	A/C
SE115	F	Leiomyosarcoma	1.7 ± 1.0	0	A/A
SE105	M	Synovial Sarcoma	1.5 ± 0.8	0	A/A
SE69*	M	Undifferentiated pleomorphic sarcoma	0.7 ± 0.7	0	A/C
SE79*	F	Undifferentiated pleomorphic sarcoma	0.6 ± 0.0	0	C/C
SE51*	F	Osteosarcoma	0.5 ± 0.5	0	A/A
SE52*	F	Leiomyosarcoma	0.4 ± 0.2	0	A/C
SE41*	M	Ewing sarcoma	0.3 ± 0.2	0	A/C
SE104	M	Undifferentiated pleomorphic sarcoma	0.3 ± 0.3	0	A/C
SE88	M	Osteosarcoma	0.3 ± 0.2	0	A/A
SE86	F	Undifferentiated pleomorphic sarcoma	0.3 ± 0.2	0	A/A
SE3	F	Undifferentiated pleomorphic sarcoma	0.2 ± 0.2	0	C/C
SE83	M	Leiomyosarcoma	0.2 ± 0.2	0	A/C
SE47*	M	Angiosarcoma	0.2 ± 0.2	0	A/A
SE77	M	Synovial sarcoma	0.0 ± 0.0	0	C/C
SE45	M	Leiomyosarcoma	0.0 ± 0.0	0	A/A

MDM4 IHC Grade 0 (<10% positive cells), Grade I (11–25% positive cells), Grade II (26–50% positive cells), Grade III (>51% positive cells). * Denotes sarcoma samples previously described in Pishas *et al*., 2014.

**Table 2:** XI-006 IC50 values of the sarcoma cell line cohort.

Cell line	Histology	*TP53* status	*MDM4* mRNA expression	XI-006 IC50 (nM, 24 hr)	XI-006 IC50 (nM, 48 hr)
STA-ET-1	ES	Wild-type	1.68 ± 0.03	376.3 ± 26.9	98.9 ± 19.5
TC252	ES	Wild-type	2.74 ± 0.04	390.2 ± 106.6	115.6 ± 6.6
SK-N-MC	ES	Truncation	0.61 ± 0.00	472.3 ± 176.4	121.8 ± 31.7
SK-ES-1	ES	Cys176Phe	0.70 ± 0.01	442.4 ± 57.5	236.0 ± 15.0
RD-ES	ES	Arg273Cys	0.32 ± 0.00	638.9 ± 142.4	299.7 ± 68.5
TC71	ES	Truncation	0.40 ± 0.02	567.9 ± 150.8	396.1 ± 78.8
WE-68	ES	Wild-type	1.09 ± 0.04	2089.3 ± 20.4	1475.7 ± 222.6
VH-64	ES	Wild-type	1.07 ± 0.01	2456.4 ± 315.1	1613.1 ± 274.4
Saos-2	OS	Null	0.17 ± 0.00	3600.2 ± 525.7	2143.0 ± 34.9
SJSA	OS	Wild-type	0.15 ± 0.01	6257.1 ± 366.5	3690.5 ± 270.2
U20S	OS	Wild-type	1.94 ± 0.04	>10 000	5416.8 ± 255.7
IMR90	LFB	Wild-type	—	8348.0 ± 231.0	6802.1 ± 696.5

ES: Ewing sarcoma, OS: Osteosarcoma, LFB: Lung fibroblast

IC_50_: Concentration of XI-006 required to induce 50% apoptosis (mean ± STDEV from two independent experiments).

*MDM4* mRNA expression determined through real-time qPCR analysis (mean ± SE from triplicate reactions).

**Table 3:** Genes identified from RNA expression profiling of Ewing and osteosarcoma cell lines treated with XI-006.

Genes significantly induced or repressed following XI-006 across all sarcoma cell lines						
Accession Number	Gene	Location	Full Name	% Change		*P* value
NM_005733	KIF20A	5q31	Kinesin family member 20A	−44.49		0.0214
NM_005063	SCD	10q24.31	Stearoyl-CoA desaturase (delta-9-desaturase)	−38.01		0.0076
NM_005896	IDH1	2q33.3	Isocitrate dehydrogenase 1 (NADP+), soluble	−37.14		0.0040
NM_013296	GPSM2	1p13.3	G-protein signalling modulator	−33.34		0.0401
NM_001013703	EIF2AK4	15q15.1	Eukaryotic translation initiation factor 2 alpha kinase	−24.35		0.0034
NM_014362	HIBCH	2q32.2	3-hydroxyisobutyryl-CoA hydrolase	−16.66		0.0134
NM_004197	STK19	6p21.3	Serine/threonine kinase 19	31.43		0.0433
NM_181706	DNAJC24	11p13	DnaJ (Hsp40) homolog, superfamily C, member 24	25.99		0.0181
NM_015666	MTG2	20q13.33	Mitochondrial ribosome-associated GTPase 2	25.18		0.0040
NM_032182	FAM175B	10q26.13	Family with sequence similarity 175, member B	24.37		0.0459
NM_144607	CYB5D1	17p13.1	Cytochrome b5 domain containing 1	20.32		0.0311
						
**Genes differentially expressed in Ewing and osteosarcoma cell lines following XI-006 treatment**						
**Accession Number**	**Gene**	**Location**	**Full Name**	**% Change Ewing sarcoma**	**% Change Osteosarcoma**	***P*** **value**
NM_020733	HEG1	3q21.2	Heart development protein with EGF-like domains 1	4.77	−73.76	0.00001
NM_005803	FLOT1	6p21.3	Flotillin	−5.44	−98.73	0.0007
NM_007124	UTRN	6q24	Utrophin	2.65	−53.96	0.0128
NM_005711	EDIL3	5q14	EGF-like repeats and discoidin I-like domains 3	−1.01	−110.0	0.0269
