# Supplementary material for: XI-006 induces potent p53-independent apoptosis in Ewing sarcoma
Source: Sci Rep. 2015 Jun 22;5:11465. doi: 10.1038/srep11465 (PMC4476092; doi:10.1038/srep11465)
Supplement: Supplementary Information [file srep11465-s1.pdf]

Supplementary Tables and Figures

XI-006 induces potent p53-independent apoptosis in Ewing sarcoma

Authors: *Kathleen I Pishas[1,2], Alaknanda Adwal[2], Susan J Neuhaus[3], Mark T Clayer[4], Gelareh Farshid[5], Alexander H Staudacher[6,7], & David F Callen[1,2]

[1] Sarcoma Research Group, Discipline of Medicine, University of Adelaide, Adelaide, Australia

[2] Cancer Therapeutics Laboratory, Discipline of Medicine, University of Adelaide, Adelaide, Australia

[3] Department of Surgery, Royal Adelaide Hospital and University of Adelaide, Adelaide, Australia

[4] Department of Orthopaedics and Trauma, Royal Adelaide Hospital, Adelaide, Australia

[5] Division of Tissue Pathology, SA Pathology, Adelaide, Australia

[6] Translational Oncology Laboratory, Centre for Cancer Biology, SA Pathology, Adelaide, Australia

[7] School of Medicine, University of Adelaide, Adelaide, Australia

Supplementary Table S1: Primer sequences utilised in this study

**Target**	**Primer sequence 5' to 3'**		**Annealing Temperature**
	**Forward**	**Reverse**	
ATM	TGGTTTGAGAAGCGATTGGC	TTCAACACCCGTAATGCCCA	65˚C
ATR	CTGATGCGTGATCAGCGAGA	ACGGCAGTCCTGTCACTCTA	65˚C
EDIL3	GAACCAACTTCAGCAGGTCCC	TAAATTCGCCTGGGCACTCA	61˚C
FLOT1	ACTGGCATTGCCCAGGTAAA	AGGGCAATGTGGGCAATCTC	63˚C
HEG1	TCCCAGAGTGGCAACTTAGC	ATCTCCGAGGTTCCACT	61˚C
IDH1	TTGGCTGCTTGCATTAAAGGTT	GTTTGGCCTGAGCTAGTTTGA	61˚C
KIF20A	CTACAAGCACCCAAGGACTCT	AGATGGAGAAGCGAATGTTT	61˚C
MDM2	TCTACAGGGACGCCATCGA	CTGATCCAACCAATCACCTGAA	61˚C
MDM4	TCTCGCTCTCGCACAGGATCACA	AACCACCAAGGCAGGCCAGCTA	61˚C
Mre11	TGCCCAGGAAAATGAAGTGGA	CAGGCCGATCACCCATACAA	61˚C
p21 (CDKN1A)	TGGACCTGGAGACTCTCAGGGTCG	TTAGGGCTTCCTCTTGGAGAAGATC	61˚C
PARP-1	AGCGAGAGCATCCCCAAGG	TCAAACATGGGCGACTGCAC	61˚C
PPIG	CAGATGCAGCTAGCAAACCGTTTG	CTCTTCAGTAGCACTTTCGGAATCAGAGG	61˚C
PUMA (BBC3)	ACGACCTCAACGCACAGTACG	TCCCATGATGAGATTGTACAGGAC	61˚C
UTRN	TTGCACTGGCAGGTGAAAGA	ACGTTGACTTGGCTGTAGGG	59˚C

Supplementary Table 2: Combination Index (CI) values obtained from XI-006/chemotherapeutic agent synergy studies

Sarcoma Cell Line	VIN IC_{50} (ng/ml)	VIN Dose (ng/ml)	VIN + XI-006 Combination Index	ActD IC_{50} (ng/ml)	ActD Dose (ng/ml)	ActD + XI-006 Combination Index	DOX IC_{50} (ng/ml)	DOX Dose (ng/ml)	DOX + XI-006 Combination Index	ETO IC50 (ng/ml)	ETO Dose (ng/ml)	ETO + XI-006 Combination Index
STA-ET-1	3.13 ± 0.70	0.098	1.078	3.80 ± 0.32	0.195	1.029	28.2 ± 1.30	1.17	1.195	592 ± 45.7	31.3	**0.936**
		0.195	1.112		0.391	**0.879**		2.34	1.190		62.5	**0.861**
		0.391	1.144		0.781	**0.794**		4.69	0.994		125	**0.891**
		0.781	1.271		1.563	**0.800**		9.38	1.009		250	**0.778**
		0.156	1.208		3.125	1.041		18.8	**0.889**		500	**0.913**
TC252	2.73 ± 0.00	0.098	**0.927**	1.74 ± 0.16	0.195	1.209	8.61 ± 0.87	0.59	**0.989**	285 ± 9.85	7.81	**0.961**
		0.195	1.026		0.391	1.513		1.17	1.025		15.6	1.078
		0.391	1.076		0.781	1.383		2.34	1.057		31.3	**0.961**
		0.781	1.157		1.563	1.034		4.69	1.007		62.5	**0.961**
		1.563	1.200								125	**0.875**
		3.125	1.867									
RD-ES	1.06 ± 0.06	0.049	1.093	8.76 ± 0.05	0.195	1.000	36.7 ± 3.81	2.34	0.997	870 ± 40.1	31.3	1.726
		0.098	1.069		0.390	**0.876**		4.69	**0.904**		62.5	1.802
		0.195	1.118		0.781	**0.902**		9.38	**0.980**		125	1.486
		0.391	1.182		1.56	1.057		18.8	**0.860**		250	1.404
		0.781	1.262		3.12	1.171		37.5	1.093		500	1.295
SK-N-MC	0.87 ± 0.04	0.024	1.061	8.72 ± 0.57	0.195	1.142	19.6 ± 1.15	1.17	1.053	559 ± 23.8	15.6	**0.985**
		0.049	1.091		0.390	0.998		2.34	1.092		31.3	**0.976**
		0.098	1.103		0.781	**0.979**		4.69	1.112		62.5	**0.932**
		0.195	1.172		1.56	**0.950**		9.38	1.063		125	**0.835**
		0.391	1.235		3.12	1.034		18.8	1.060		250	**0.753**
WE-68	2.31 ± 0.02	0.049	1.063	1.72 ± 0.02	0.049	**0.899**	23.7 ± 0.27	0.59	1.050	1000 ± 115	31.3	1.066
		0.098	1.084		0.098	**0.969**		1.17	**0.864**		62.5	1.051
		0.195	1.151		0.195	1.026		2.34	**0.972**		125	1.048
		0.391	1.076		0.391	1.161		4.69	**0.976**		250	1.035
		0.781	1.333		0.781	**0.945**		9.38	**0.898**		500	1.007
		1.563	1.063		1.563	1.512		18.8	**0.965**		1000	1.624

VIN: Vincristine, ActD: Actinomycin D, DOX: Doxorubicin, ETO: Etoposide. CI value of <1, =1 and >1 indicates synergistic, additive and antagonistic effects respectively. CI numbers in bold denote synergism.

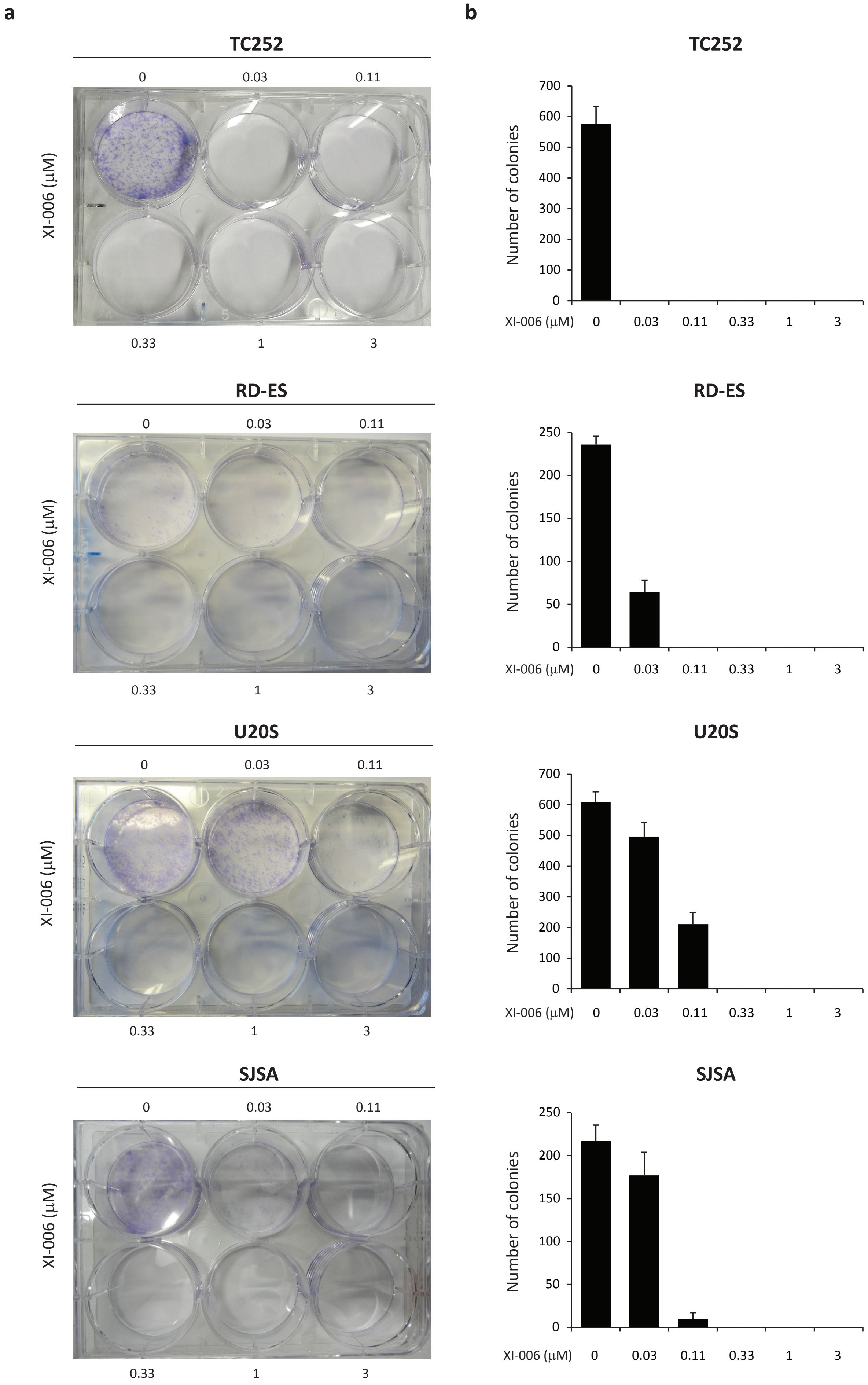

a
b
TC252
RD-ES
U20S
SJSA
XI-006 (μM)
0
0.03
0.11
0.33
1
3
Number of colonies
0
100
200
300
400
500
600
700
50
150
250

c

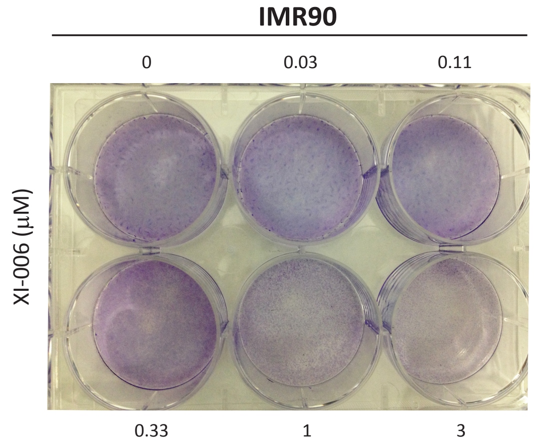

Supplementary Figure 1: *XI-006 reduces cellular proliferation specifically in cancer cell lines*

(a) Ewing (TC252, RD-ES) and osteosarcoma (U20S and SJSA) cell lines were seeded ($3x10^3$ cells/well) in 6-well plates in the presence or absence of XI-006 (0.03, 0.11, 0.33, 1, 3μM). Cells were fixed in methanol (5mins) and stained with giemsa (Sigma) (50 mins) 10 days post seeding. (b) Quantification of colonies from cells treated as in (a). Data represents mean ± STDEV from two independent experiments. (c) Representative image of IMR90 cells (normal human fibroblasts) treated as in (a).

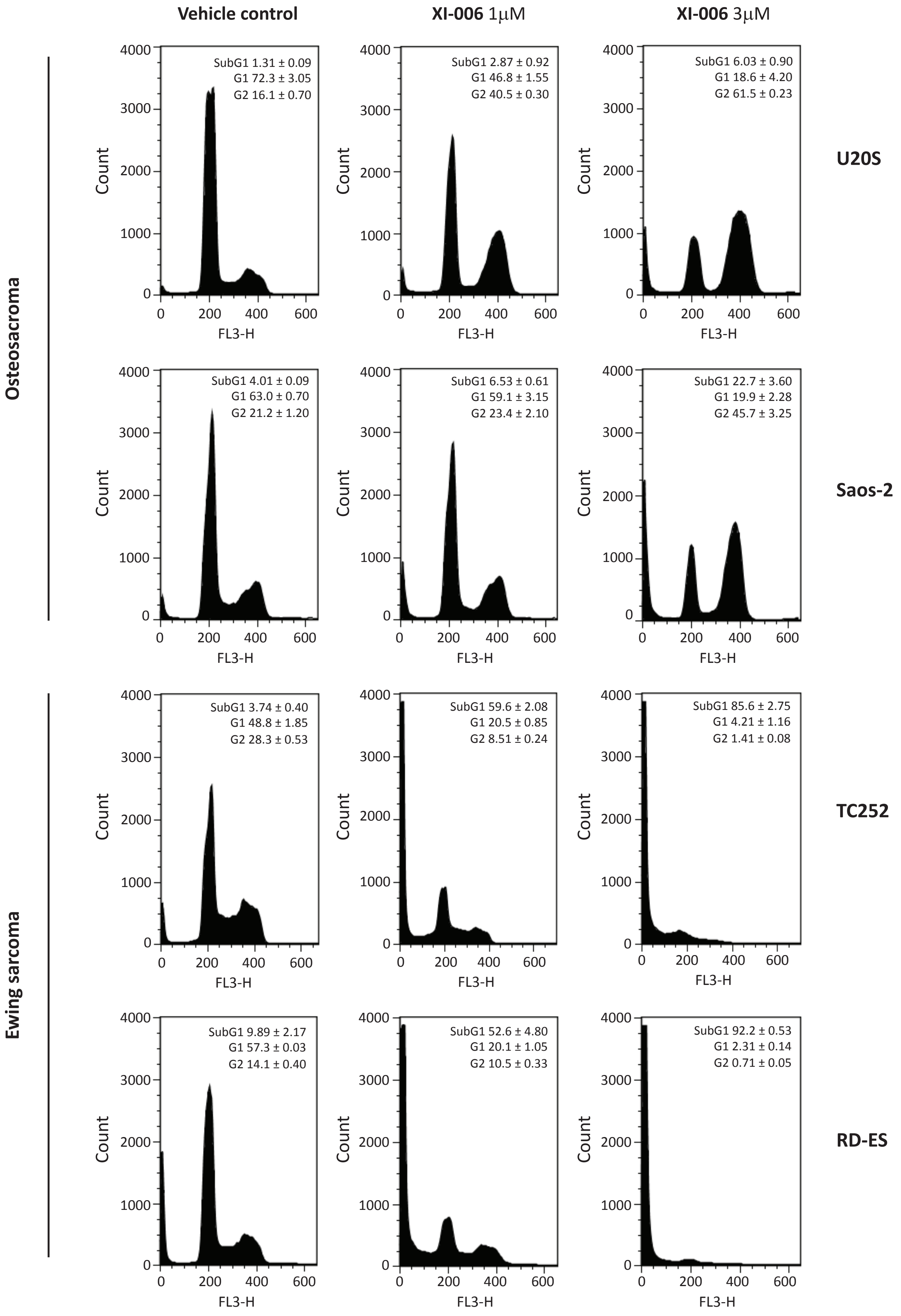

Supplementary Figure 2. *XI-006 induces apoptosis specifically in Ewing sarcoma cells*

Ewing (TC252, RD-ES) and osteosarcoma (U20S, Soas-2) cells were treated with XI-006 (1μM, 3 μM) or vehicle control for 48hrs. Cells were fixed, stained with propidium iodide, and analyzed by flow cytometry for DNA content. Numbers inserted in graphs indicate percentage of cells at different stages of the cell cycle (mean ± STDEV from two independent experiments). 60,000 PI stained cells were analysed for DNA content.

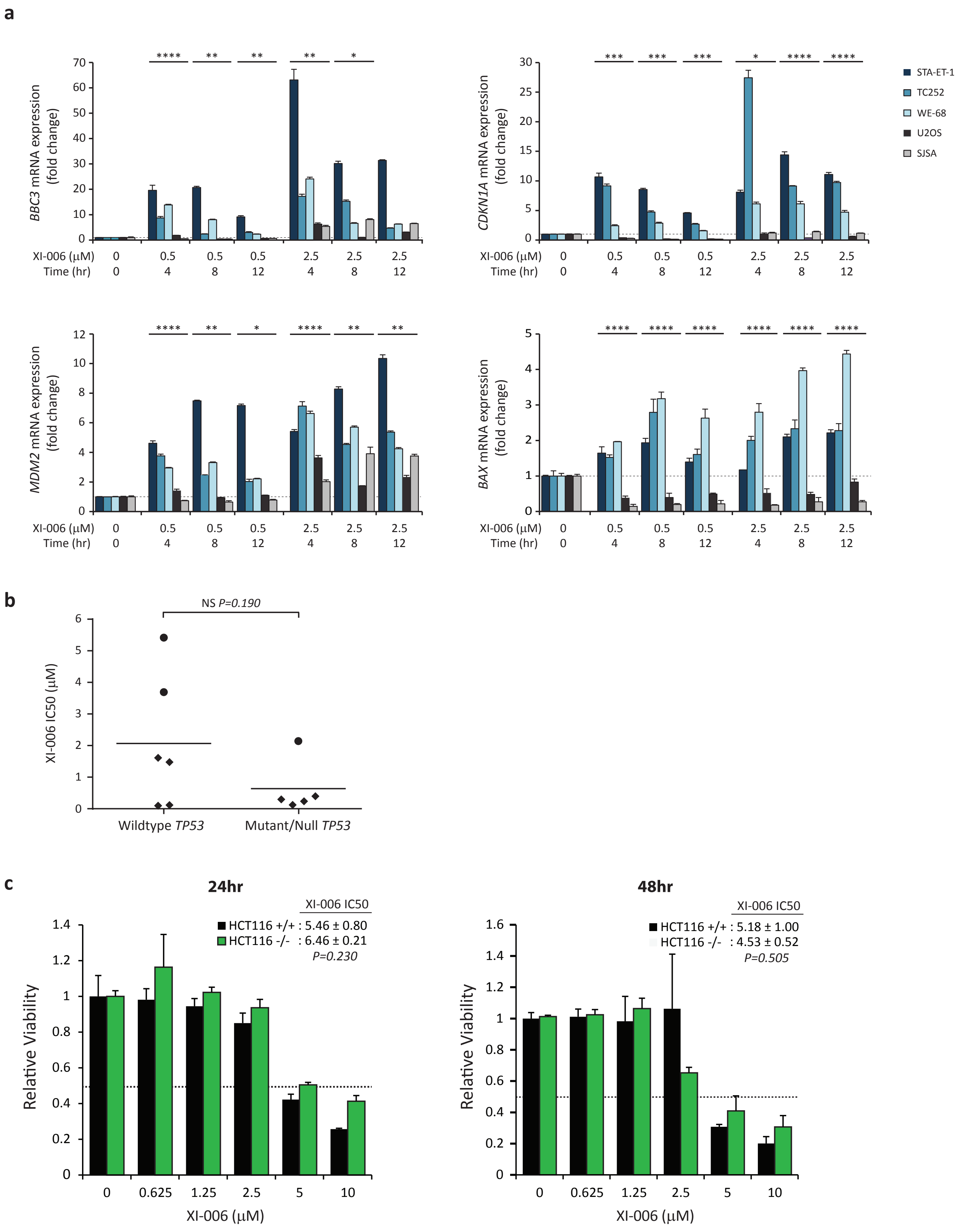

Supplementary Figure 3: *XI-006 induces expression of p53 target genes specifically in Ewing sarcoma cell lines*

(a) Wild-type Ewing (STA-ET-1, TC252,WE -68) and osteosarcoma (U20S, SJSA) cell lines were treated with XI-006 (0, 2.5 and 5µM) for the indicated times. mRNA expression levels of *TP53* target genes (*BBC3, CDKN1A, MDM2, BAX*) was determined through real-time (qPCR) analysis. Data represents mean expression (fold change) ± SE from triplicate reactions. Asterisk denotes statistical significance in target gene expression (Ewing versus osteosarcoma cell lines) (**P<0.05, **P<0.01, ***P<0.001, **** P<0.0001*). (b) Lack of correlation between XI-006 apoptotic 48hr IC50 values and *TP53* status (wild-type versus mutant/null). ◆●Denotes Ewing sarcoma and osteosarcoma cell lines respectively. (c) Relative viability of *TP53* wild-type (+/+) and null (-/-) HCT116 isogenic cell lines following treatment with XI-006 for 24 and 48hrs. Data represents mean ± STDEV from duplicate reactions. Viability determined through Cell Titer-Glo assays.

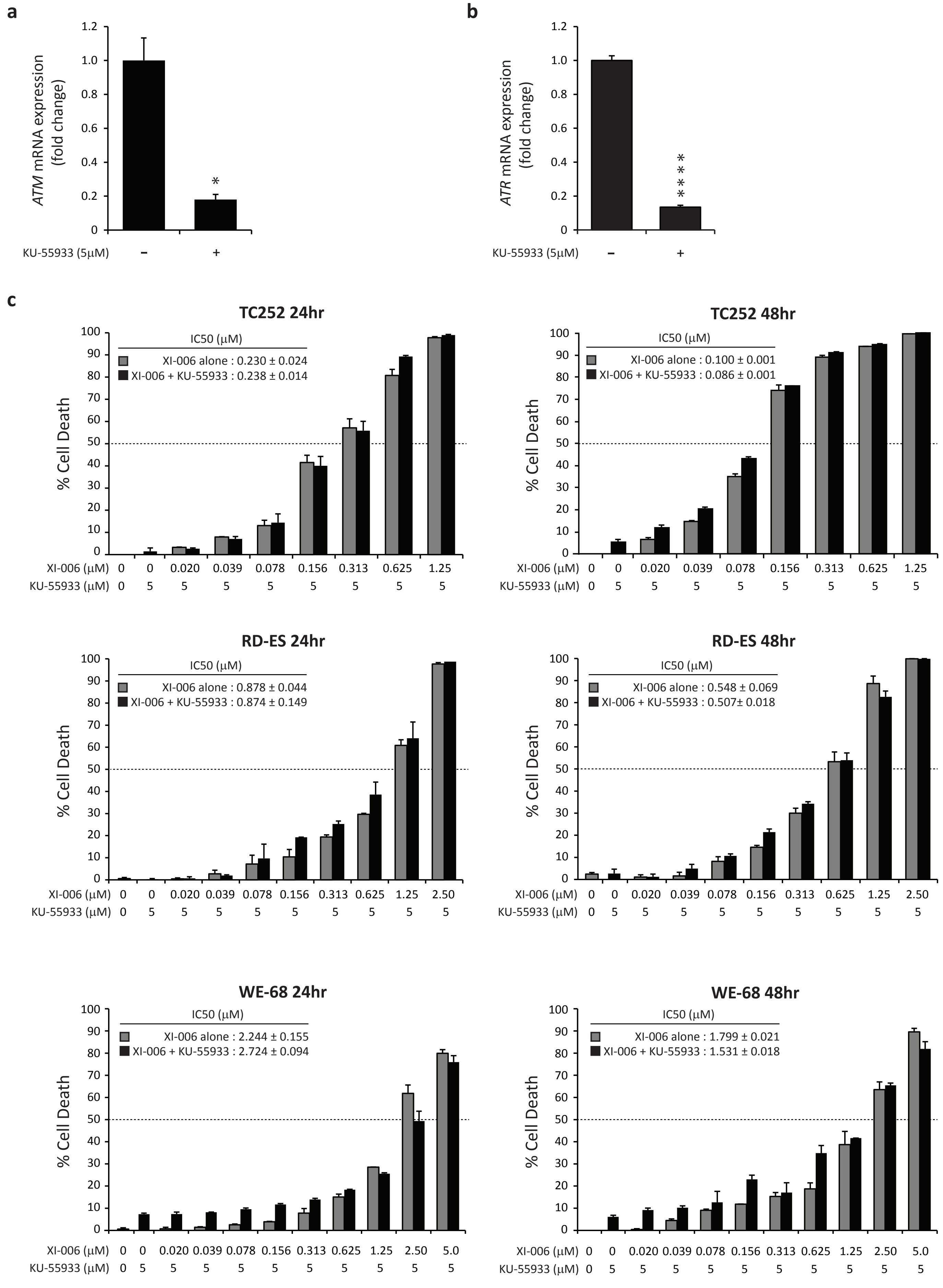

Supplementary Figure 4: *Inhibition of ATM does not supress XI-006 cytotoxicity*

Real-time qPCR analysis of (a) *ATM* and (b) *ATR* mRNA expression following treatment with the ATM inhibitor KU-55933 (5μM) for 6hrs. Data represents mean expression (fold change) ± SE from triplicate reactions. Asterisk denotes statistically difference compared to vehicle control treated cells (**P<0.05, ****P<0.0001*). (c) TC252, RDES and WE-68 were pre-treated with KU-55933 (5μM) or vehicle control (DMSO) for 2hrs, prior to the addition of XI-006 (0-5μM). Cell viability was determined through 7AAD staining (24 and 48hrs post XI-006 treatment) and analysed by flow cytometry. Data represents average percentage cell death ± STDEV from duplicate reactions.

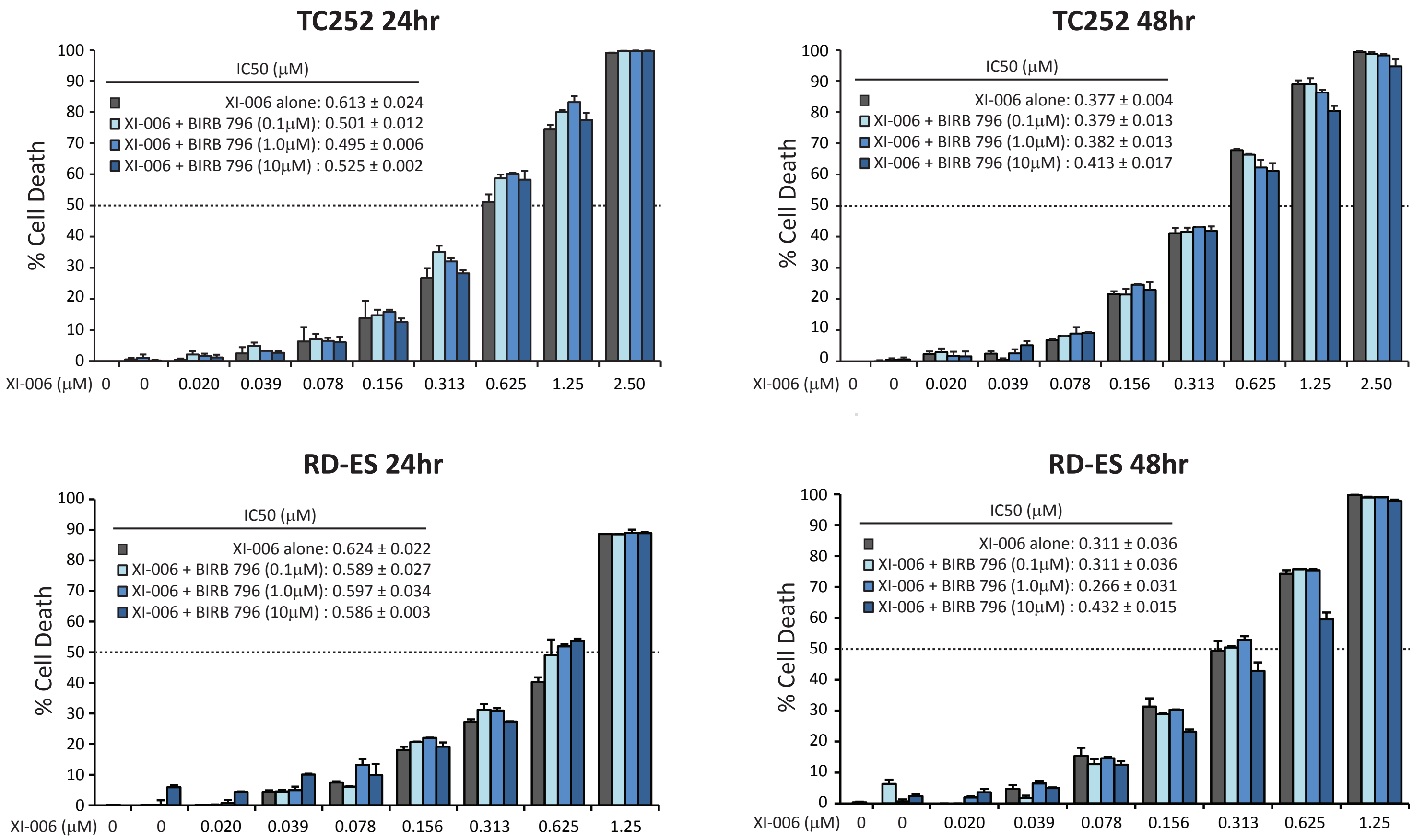

Supplementary Figure 5: *Inhibition of p38 MAPKs has no effect on XI-006 sensitivity*

TC252 and RD-ES cells were pre-treated with BIRB 796 (0.1µM, 1 µM and 10µM) or vehicle control (DMSO) for 2hrs, prior to the addition of XI-006 (0-2.5µM). Percentage cell death was determined through 7AAD staining (24 and 48hrs post XI-006 treatment) and analysed by flow cytometry. Data represents average percentage cell death ± STDEV from duplicate reactions.

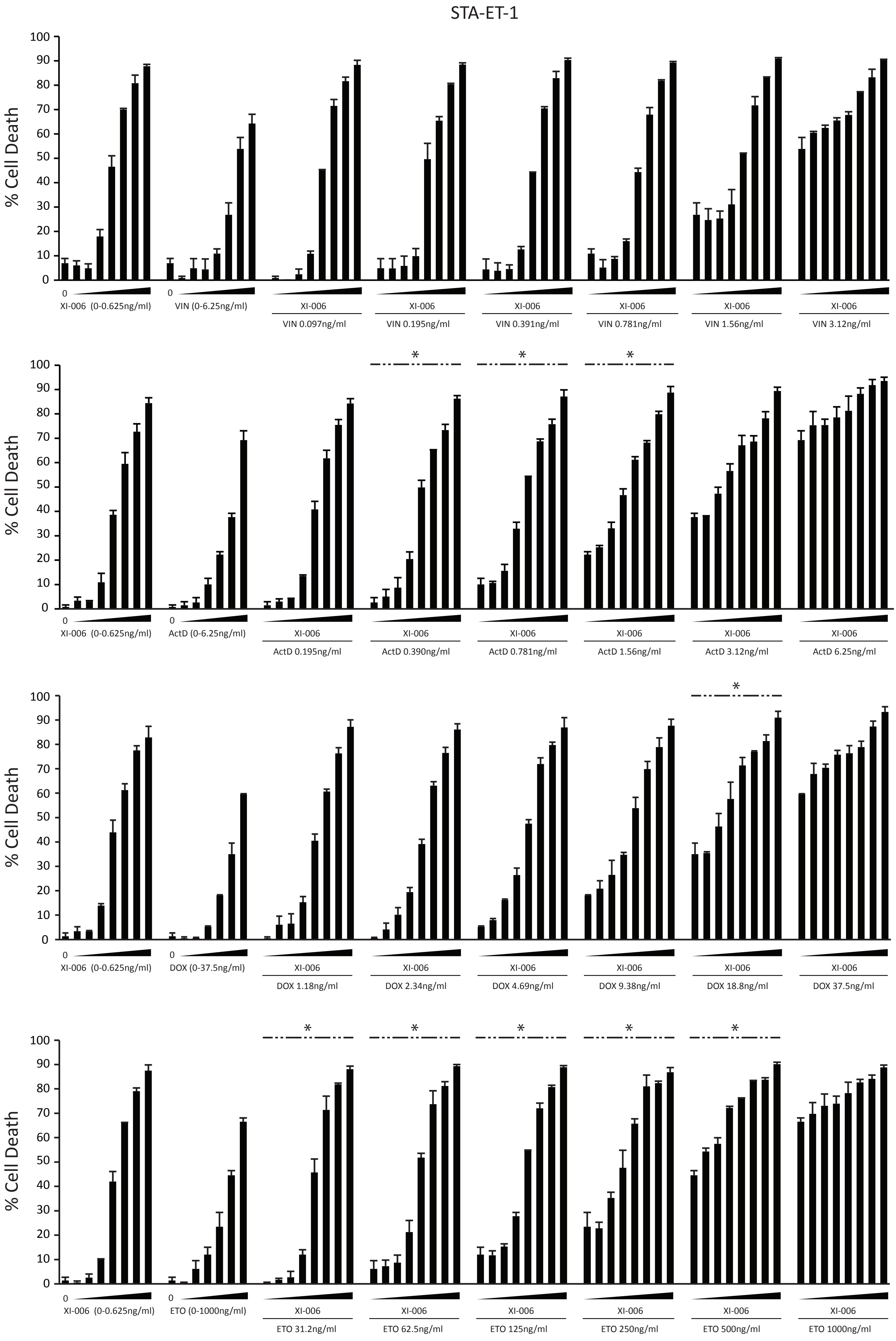

STA-ET-1
% Cell Death
XI-006 (0-0.625ng/ml)
VIN (0-6.25ng/ml)
VIN 0.097ng/ml
VIN 0.195ng/ml
VIN 0.391ng/ml
VIN 0.781ng/ml
VIN 1.56ng/ml
VIN 3.12ng/ml
ActD (0-6.25ng/ml)
ActD 0.195ng/ml
ActD 0.390ng/ml
ActD 0.781ng/ml
ActD 1.56ng/ml
ActD 3.12ng/ml
ActD 6.25ng/ml
DOX (0-37.5ng/ml)
DOX 1.18ng/ml
DOX 2.34ng/ml
DOX 4.69ng/ml
DOX 9.38ng/ml
DOX 18.8ng/ml
DOX 37.5ng/ml
ETO (0-1000ng/ml)
ETO 31.2ng/ml
ETO 62.5ng/ml
ETO 125ng/ml
ETO 250ng/ml
ETO 500ng/ml
ETO 1000ng/ml

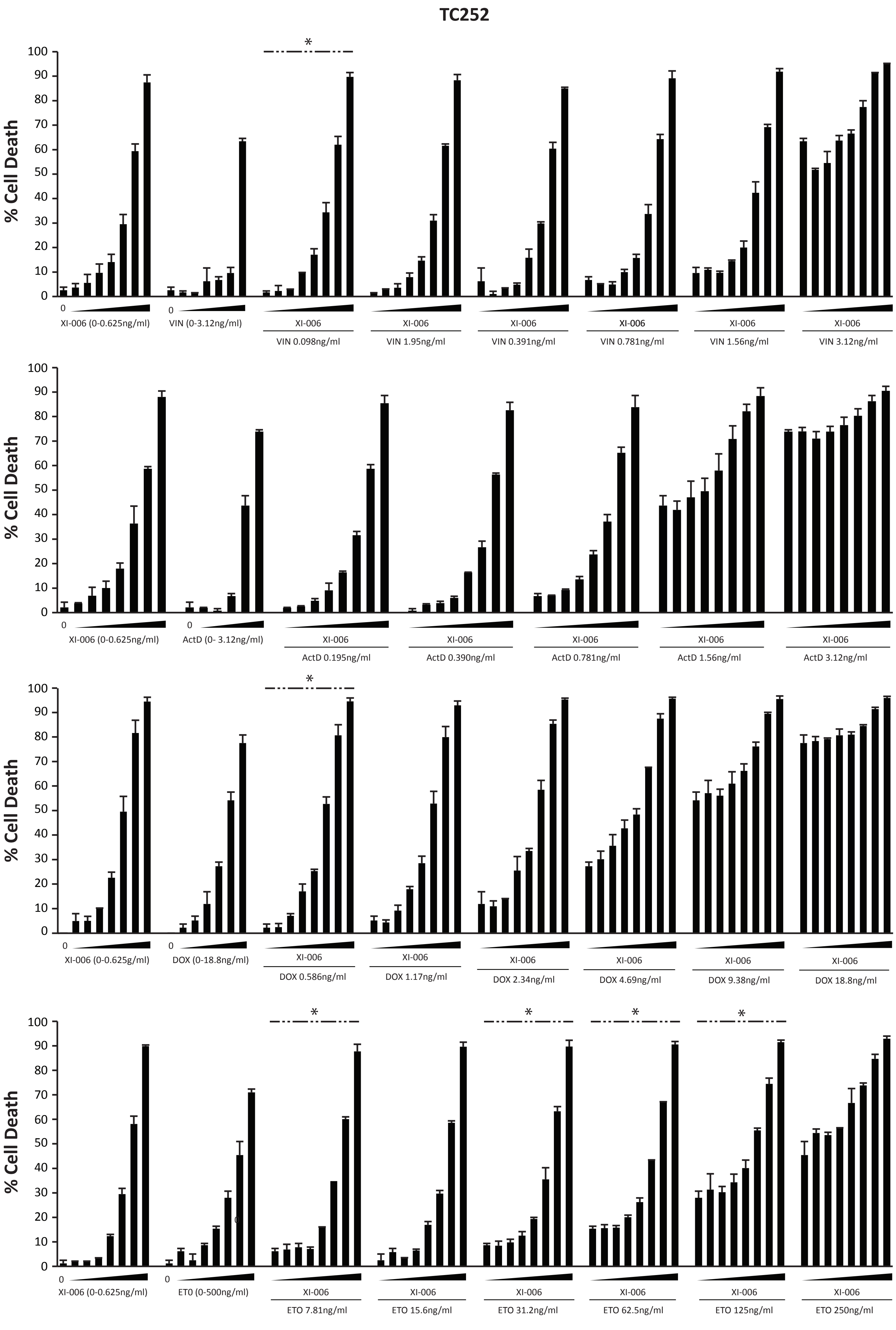
TC252
% Cell Death
0
10
20
30
40
50
60
70
80
90
100
*
XI-006 (0-0.625ng/ml)
VIN (0-3.12ng/ml)
XI-006
VIN 0.098ng/ml
VIN 1.95ng/ml
VIN 0.391ng/ml
VIN 0.781ng/ml
VIN 1.56ng/ml
VIN 3.12ng/ml
ActD (0- 3.12ng/ml)
ActD 0.195ng/ml
ActD 0.390ng/ml
ActD 0.781ng/ml
ActD 1.56ng/ml
ActD 3.12ng/ml
XI-006 (0-0.625g/ml)
DOX (0-18.8ng/ml)
DOX 0.586ng/ml
DOX 1.17ng/ml
DOX 2.34ng/ml
DOX 4.69ng/ml
DOX 9.38ng/ml
DOX 18.8ng/ml
ET0 (0-500ng/ml)
ETO 7.81ng/ml
ETO 15.6ng/ml
ETO 31.2ng/ml
ETO 62.5ng/ml
ETO 125ng/ml
ETO 250ng/ml

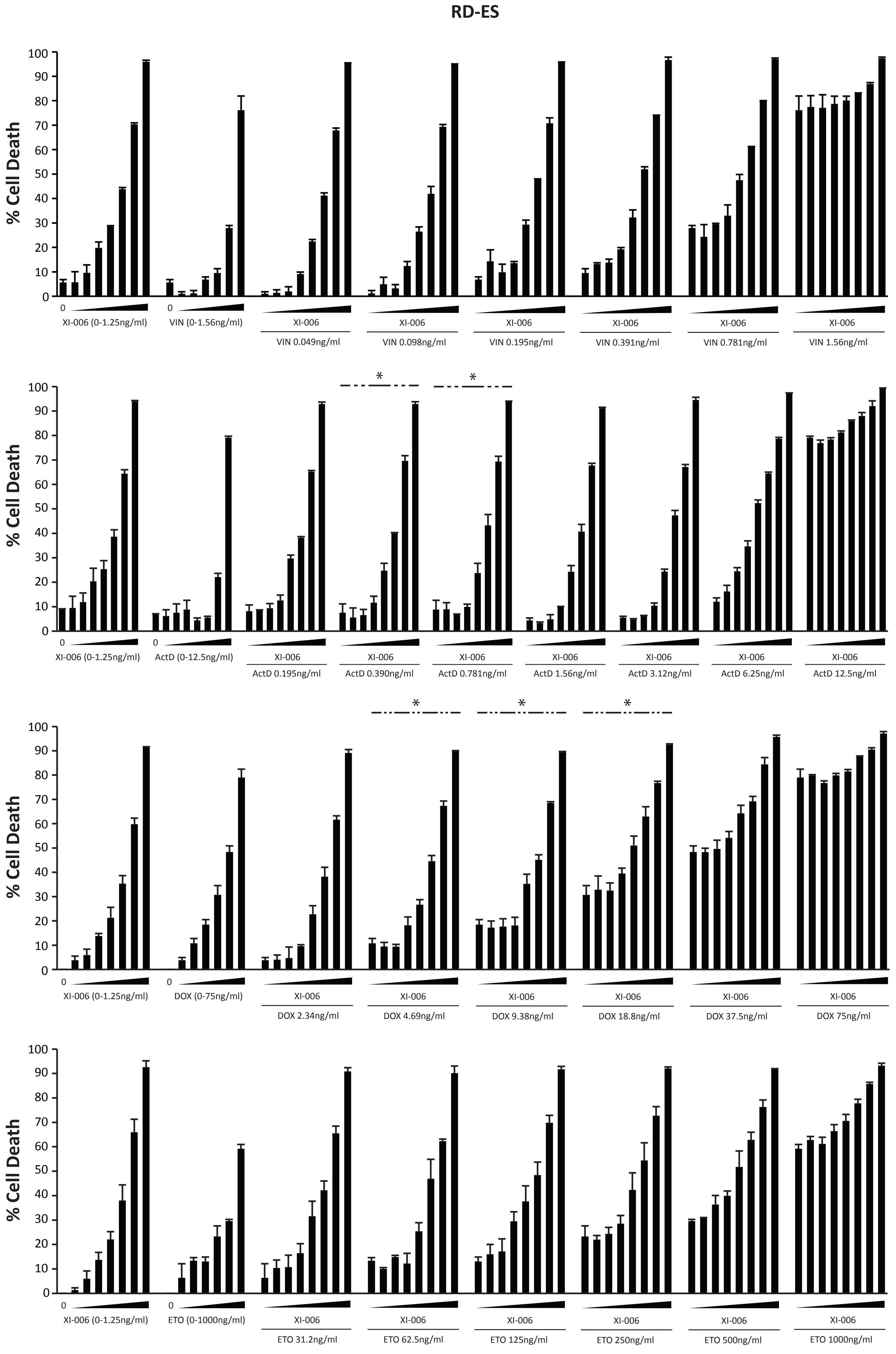
RD-ES
% Cell Death
0
XI-006 (0-1.25ng/ml)
VIN (0-1.56ng/ml)
XI-006
VIN 0.049ng/ml
VIN 0.098ng/ml
VIN 0.195ng/ml
VIN 0.391ng/ml
VIN 0.781ng/ml
VIN 1.56ng/ml
ActD (0-12.5ng/ml)
ActD 0.195ng/ml
ActD 0.390ng/ml
ActD 0.781ng/ml
ActD 1.56ng/ml
ActD 3.12ng/ml
ActD 6.25ng/ml
ActD 12.5ng/ml
DOX (0-75ng/ml)
DOX 2.34ng/ml
DOX 4.69ng/ml
DOX 9.38ng/ml
DOX 18.8ng/ml
DOX 37.5ng/ml
DOX 75ng/ml
ETO (0-1000ng/ml)
ETO 31.2ng/ml
ETO 62.5ng/ml
ETO 125ng/ml
ETO 250ng/ml
ETO 500ng/ml
ETO 1000ng/ml
*

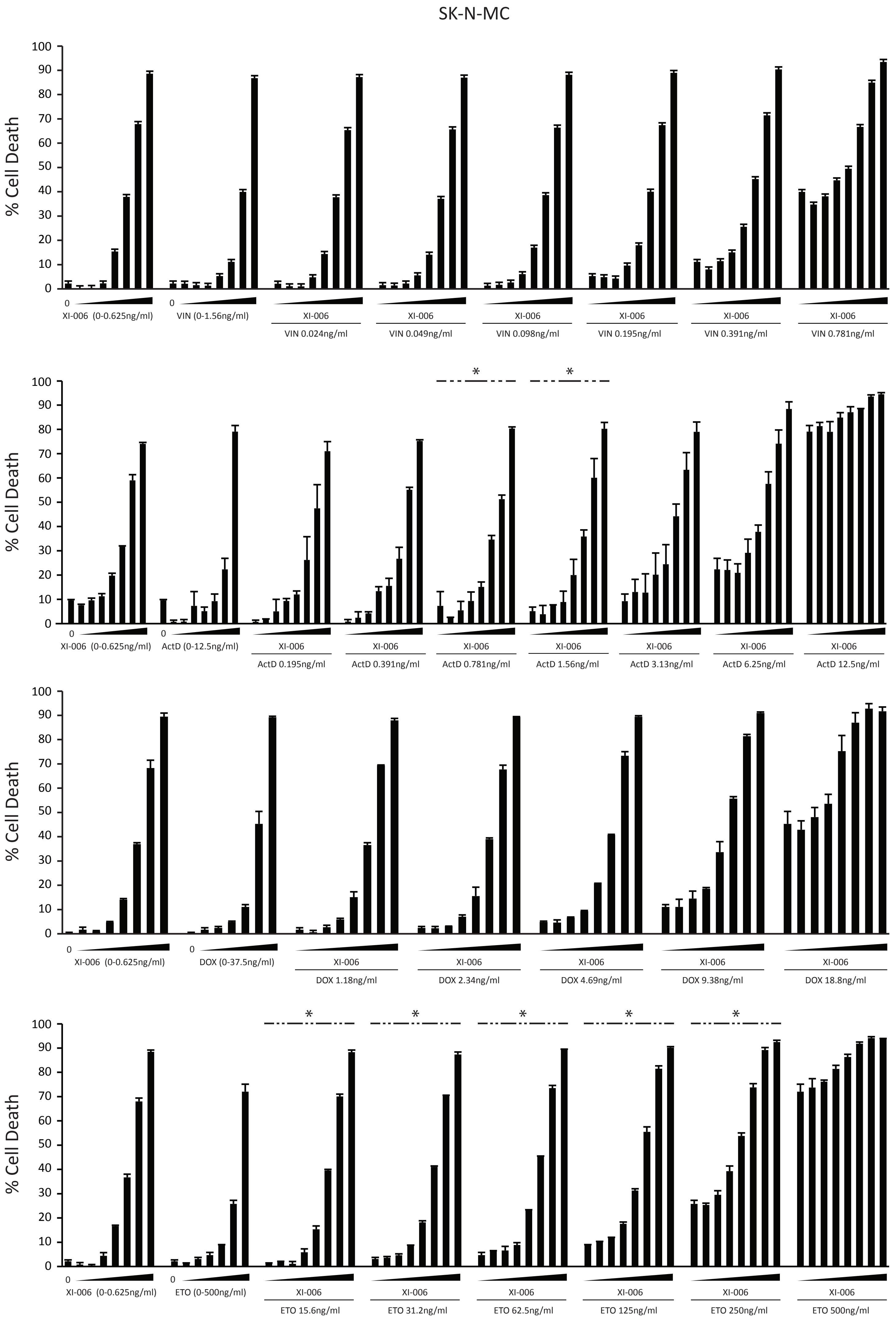
SK-N-MC
% Cell Death
XI-006 (0-0.625ng/ml)
VIN (0-1.56ng/ml)
XI-006
VIN 0.024ng/ml
VIN 0.049ng/ml
VIN 0.098ng/ml
VIN 0.195ng/ml
VIN 0.391ng/ml
VIN 0.781ng/ml
ActD (0-12.5ng/ml)
ActD 0.195ng/ml
ActD 0.391ng/ml
ActD 0.781ng/ml
ActD 1.56ng/ml
ActD 3.13ng/ml
ActD 6.25ng/ml
ActD 12.5ng/ml
DOX (0-37.5ng/ml)
DOX 1.18ng/ml
DOX 2.34ng/ml
DOX 4.69ng/ml
DOX 9.38ng/ml
DOX 18.8ng/ml
ETO (0-500ng/ml)
ETO 15.6ng/ml
ETO 31.2ng/ml
ETO 62.5ng/ml
ETO 125ng/ml
ETO 250ng/ml
ETO 500ng/ml

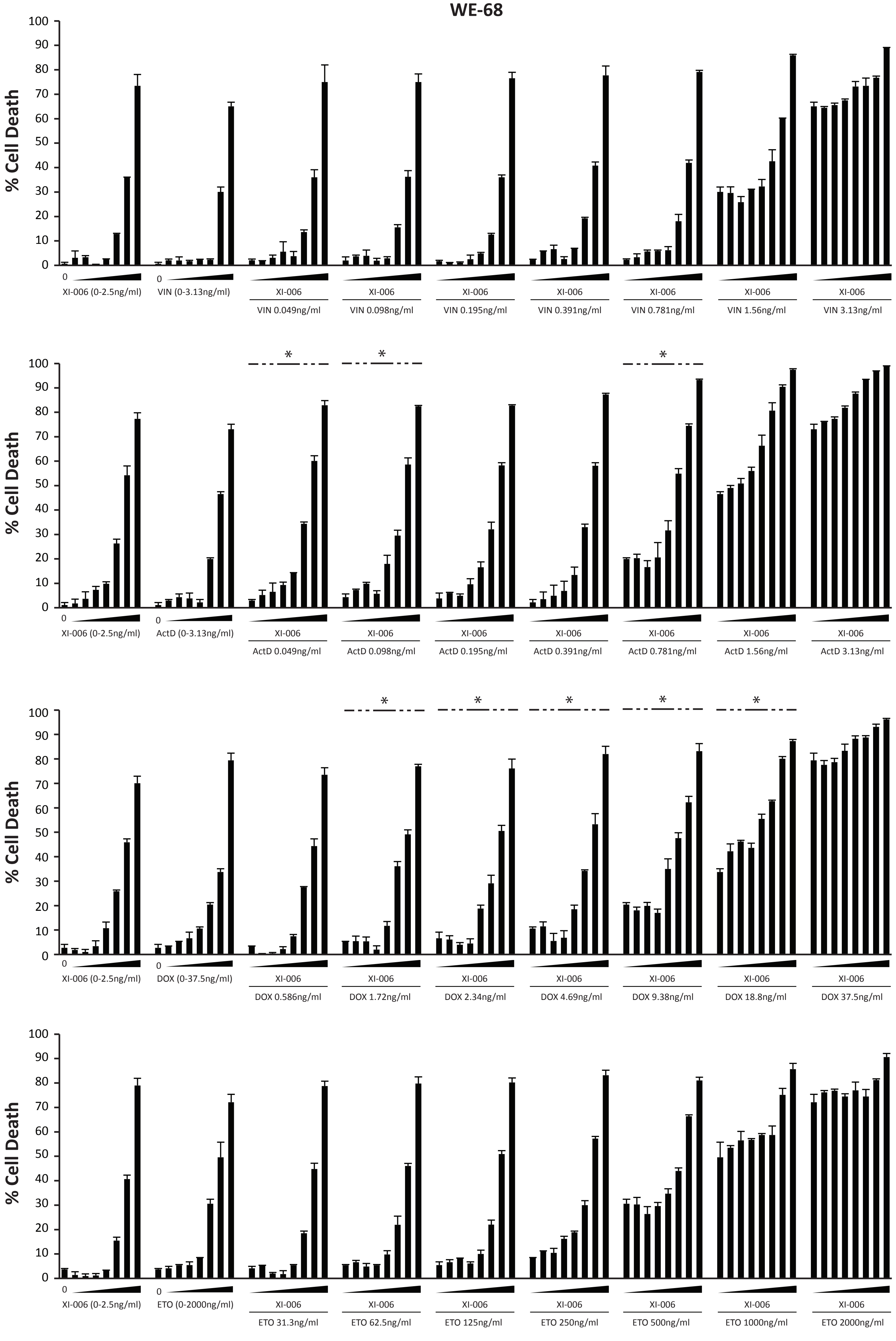
WE-68
% Cell Death
XI-006 (0-2.5ng/ml)
VIN (0-3.13ng/ml)
XI-006
VIN 0.049ng/ml
VIN 0.098ng/ml
VIN 0.195ng/ml
VIN 0.391ng/ml
VIN 0.781ng/ml
VIN 1.56ng/ml
VIN 3.13ng/ml
ActD (0-3.13ng/ml)
ActD 0.049ng/ml
ActD 0.098ng/ml
ActD 0.195ng/ml
ActD 0.391ng/ml
ActD 0.781ng/ml
ActD 1.56ng/ml
ActD 3.13ng/ml
DOX (0-37.5ng/ml)
DOX 0.586ng/ml
DOX 1.72ng/ml
DOX 2.34ng/ml
DOX 4.69ng/ml
DOX 9.38ng/ml
DOX 18.8ng/ml
DOX 37.5ng/ml
ETO (0-2000ng/ml)
ETO 31.3ng/ml
ETO 62.5ng/ml
ETO 125ng/ml
ETO 250ng/ml
ETO 500ng/ml
ETO 1000ng/ml
ETO 2000ng/ml

Supplementary Figure 6: XI-006 synergises with etoposide, doxorubicin, and actinomycin D but not vincristine

STA-ET-1, TC252, RD-ES, SK-N-MC and WE-68 cells were treated with the chemotherapeutic agents vincristine (VIN), actinomycin D (ActD), doxorubicin (DOX) and Etoposide (ETO) alone or in the presence of XI-006 (indicated concentrations) for 48hrs. Cell viability was determined through 7AAD staining and analysed by flow cytometry. Data represents average percentage cell death ± STDEV from duplicate reactions. Asterisk denotes a Combination Index (CI) of <1, indicating synergy.

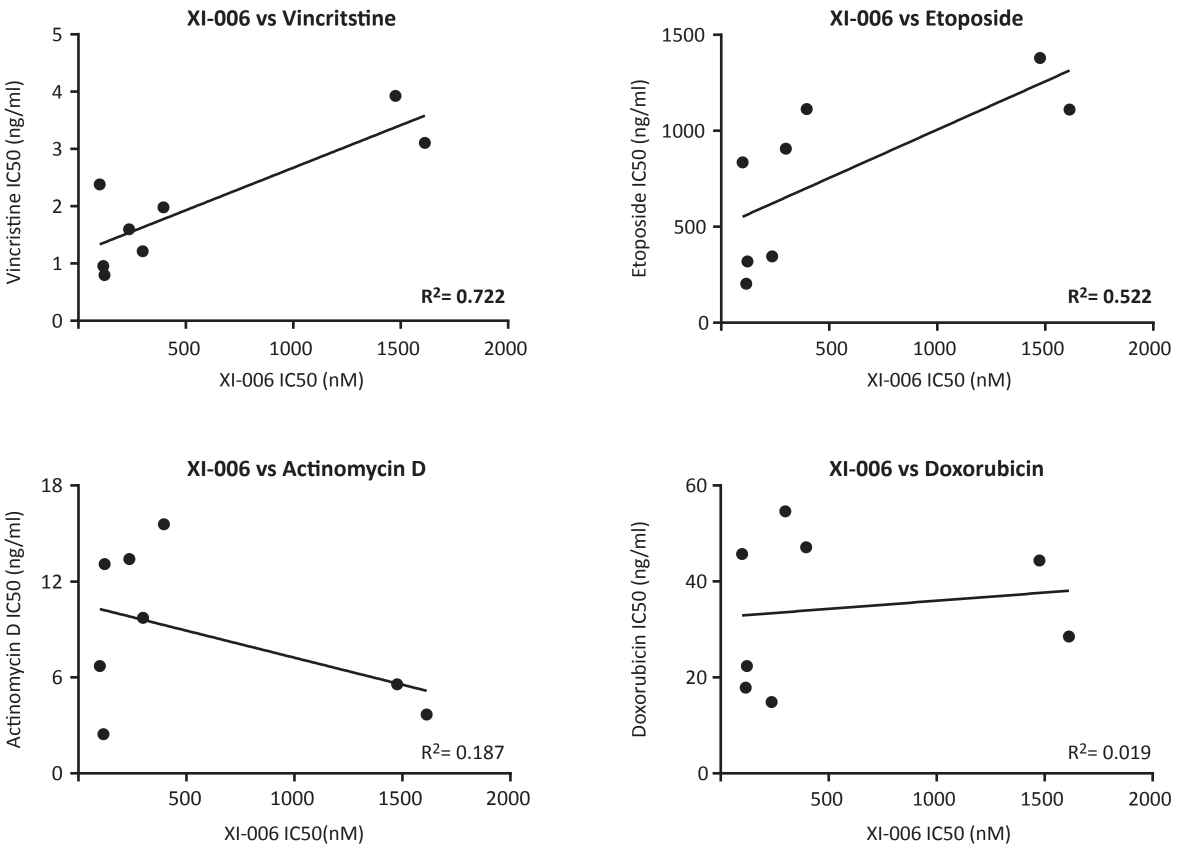

Supplementary Figure 7: *XI-006 sensitivity correlates with vincristine sensitivity in Ewing sarcoma cell lines*

Correlation between XI-006 IC50 values and chemotherapeutic (vincristine, actinomycin D, doxorubicin, etoposide) IC50 values. All Ewing sarcoma cell lines (n=8) were treated with chemotherapeutic agents or XI-006 for 48hrs. Cell viability was determined through 7AAD staining and analysed by flow cytometry. Data represents mean apoptotic IC50 values (duplicate wells).

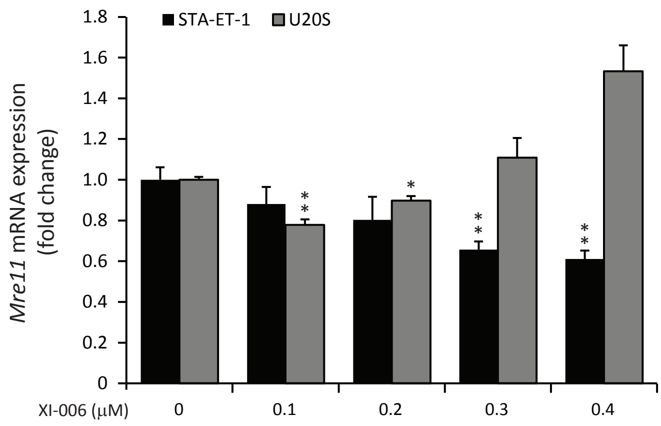

Supplementary Figure 8: *Low micro-molar doses of XI-006 reduces Mre11 mRNA expression*

Mre11 mRNA expression levels were determined through real-time qPCR analysis from STA-ET-1 and U20S cells treated with XI-006 (0.1, 0.2, 0.3 and 0.4μM) or vehicle control for 6hrs. Data represents mean expression (fold change) ± SE from triplicate reactions. Asterisk denotes statistical significant reduction in expression compared to vehicle control (*P<0.05, **P<0.01)

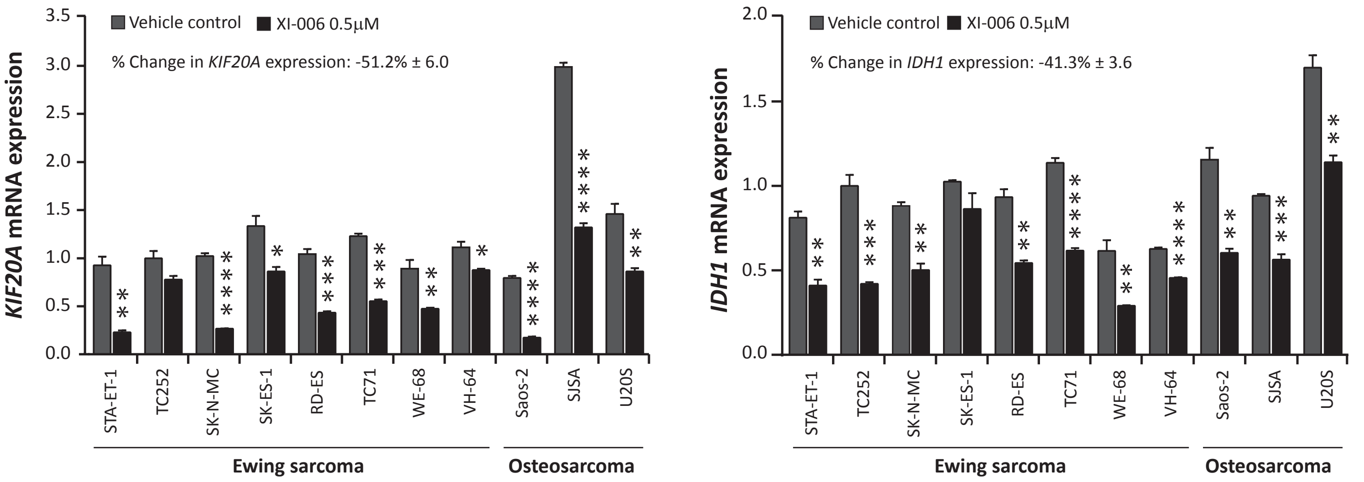

Supplementary Figure 9: *KIF20A* and *IDH1* are repressed following XI-006 treatment

Real-time qPCR analysis of *KIF20A* and *IDH1* expression levels following 4hr XI-006 (0.5μM) or vehicle control treatment. Data represents mean ± SE from triplicate reactions. Asterisk denotes statistical significance compared to vehicle control (*P<0.05, **P<0.01, ***P<0.001, ****P<0.0001).

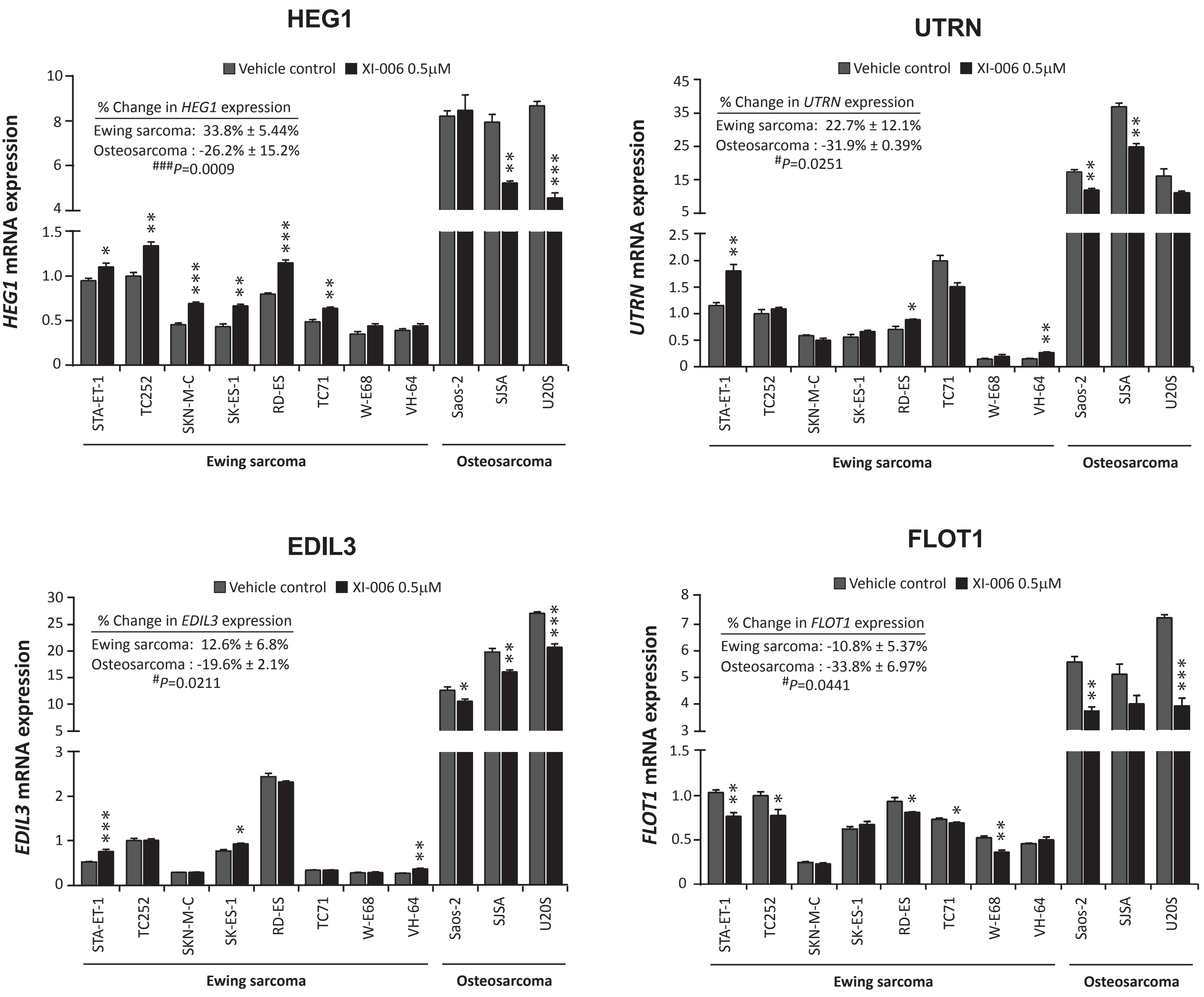

Supplementary Figure 10: *EDIL3, UTRN, HEG1* and *FLOT1* expression levels following XI-006 treatment

Real-time qPCR analysis of *EDIL3*, *UTRN*, *HEG1* and *FLOT1* expression levels following 4hr XI-006 treatment (0.5µM). Data represents mean ± SE from triplicate reactions. (*) denotes statistical significance in expression compared to vehicle control (*P<0.05, **P<0.01, ***P<0.001). (#) denotes significant difference in % change in expression, Ewing sarcoma cell lines versus osteosarcoma cell lines ($^{\#}$ P<0.05, $^{\#\#\#}$ P<0.001)
